# The Sac10b homolog from *Sulfolobus islandicus* is an RNA chaperone

**DOI:** 10.1093/nar/gkaa656

**Published:** 2020-08-06

**Authors:** Ningning Zhang, Li Guo, Li Huang

**Affiliations:** State Key Laboratory of Microbial Resources, Institute of Microbiology, Chinese Academy of Sciences, No.1 Beichen West Road, Chaoyang District, Beijing 100101, China; College of Life Sciences, University of Chinese Academy of Sciences, No.19A Yuquan Road, Shijingshan District, Beijing 100049, China; State Key Laboratory of Microbial Resources, Institute of Microbiology, Chinese Academy of Sciences, No.1 Beichen West Road, Chaoyang District, Beijing 100101, China; State Key Laboratory of Microbial Resources, Institute of Microbiology, Chinese Academy of Sciences, No.1 Beichen West Road, Chaoyang District, Beijing 100101, China; College of Life Sciences, University of Chinese Academy of Sciences, No.19A Yuquan Road, Shijingshan District, Beijing 100049, China

## Abstract

Nucleic acid-binding proteins of the Sac10b family, also known as Alba, are widely distributed in Archaea. However, the physiological roles of these proteins have yet to be clarified. Here, we show that Sis10b, a member of the Sac10b family from the hyperthermophilic archaeon *Sulfolobus islandicus*, was active in RNA strand exchange, duplex RNA unwinding *in vitro* and RNA unfolding in a heterologous host cell. This protein exhibited temperature-dependent binding preference for ssRNA over dsRNA and was more efficient in RNA unwinding and RNA unfolding at elevated temperatures. Notably, alanine substitution of a highly conserved basic residue (K) at position 17 in Sis10b drastically reduced the ability of this protein to catalyse RNA strand exchange and RNA unwinding. Additionally, the preferential binding of Sis10b to ssRNA also depended on the presence of K17 or R17. Furthermore, normal growth was restored to a slow-growing Sis10b knockdown mutant by overproducing wild-type Sis10b but not by overproducing K17A in this mutant strain. Our results indicate that Sis10b is an RNA chaperone that likely functions most efficiently at temperatures optimal for the growth of *S. islandicus*, and K17 is essential for the chaperone activity of the protein.

## INTRODUCTION

Nucleic acid-binding proteins of the Sac10b family (also referred to as Alba) are widely distributed in Archaea with very few exceptions (e.g., *Halobacterium*) (1). Sac10b homologs are also present in Eukarya, including yeast, kinetoplastids, apicomplexans, plants and animals (1). Members of the Sac10b family from *Sulfolobus* species were initially shown to bind cooperatively to double-stranded (ds) DNA without apparent sequence preferences and thus have been canonically regarded as a chromatin protein (2–6). These proteins exist as a dimer in solution and are capable of bridging and rigidifying DNA, as well as constraining negative DNA supercoils in a temperature-dependent manner *in vitro* (6,7). Furthermore, Ssh10b, a Sac10b homologue from *S. shibatae*, was shown to bind RNA as well as DNA *in vitro* and to be associated exclusively with RNA (e.g. rRNA and mRNA) *in vivo* (8). RNA binding was also observed with Pho10b, a Sac10b homologue from *Pyrococcus horikoshii*, using a gel shift assay (9). Using comparative genomics and sequence profile searches, Aravind and colleagues (1) were able to unify the archaeal Sac10b proteins with the eukaryotic RNase P/MRP subunits Pop7/Rpp20 and Rpp25, and the ciliate protein Mdp2. The eukaryotic homologs of the Sac10b proteins are principally involved in RNA metabolism (10–18). More recently, Ssh10b was shown to destabilize base pairing of RNA secondary structures by RNase T1 footprinting, prompting speculation that this protein might serve a role in biological processes involving RNA, such as translation, that require destabilization of RNA secondary structures (19).

The 3D structures of several Sac10b homologs alone or in complex with dsDNA or dsRNA have been solved by X-ray crystallography or NMR spectroscopy (4,9,19–26). All of these proteins exhibit very similar structures, with a β1–α1–β2–α2–β3–β4 connection. In an Ssh10b–dsRNA co-crystal, Ssh10b binds two RNA strands symmetrically as a tetramer with each dimer bound asymmetrically to a single RNA strand in the major groove (19). Four positively charged amino acid residues (K16, K17, R42 and R44) and Y22 are involved in RNA binding. There are two dimer-dimer interfaces responsible for intra- and inter-dsRNA fibre protein–protein interactions, respectively. The intra-fibre dimer-dimer interface consists of four main chain hydrogen bonds between two parallel β-strands and two hydrogen bonds between amino acid residues from the interacting dimers. The inter-fibre dimer–dimer interface involves a hydrophobic patch (M20, L24, L27 and F60), which is highly conserved across the Sac10b family.

Although it is known that proteins of the Sac10b family interact with RNA, the potential role of these proteins in biological processes involving RNA is unclear. In addition, the structure-function relationships of these proteins have been studied almost exclusively *in vitro*. Thus, the physiological relevance of the biochemical properties of the Sac10b family remains elusive. In this report, we show that Sis10b, a Sac10b homolog from *S. islandicus*, is an RNA chaperone active in RNA strand exchange, duplex RNA unwinding *in vitro* and RNA unfolding in a heterologous host cell. We constructed an *S. islandicus* mutant strain containing Sis10b, an essential protein, at a reduced expression level, and tested the effect of various mutations in Sis10b on the growth of these cells using complementation assays. We further demonstrate that lysine 17 is required for the ssRNA binding capacity of this protein and the survival of the organism.

## MATERIALS AND METHODS

### Overexpression and purification of Sis10b and its mutants

Genes encoding wild-type and N-terminally truncated Sis10b (ΔN12) were amplified by PCR from the genomic DNA from *S. islandicus* REY15A and cloned into the NdeI and XhoI sites of the expression plasmid pET30a (for primers sequences, see Supplementary Table S1). Plasmids encoding mutant Sis10b proteins, i.e., K16A, K17A, K17R, K16N/R44I, K16N/R83T and R44I/R83T, were prepared by site-directed mutagenesis using pET30a-*sis10b* as a template. Genes encoding K16N/R44I/R83T and M20E/L24E/L27E/F60E were synthesized at TsingKe Biotech, Beijing, China, and cloned into the NdeI and XhoI sites of pET30a. The wild-type and mutant *sis10b* sequences were then verified by DNA sequencing. These mutant proteins were overexpressed and purified as previously described (6). The concentrations of the purified proteins were determined by the Lowry method using bovine serum albumin (BSA) as a standard (27).

### Electrophoretic mobility shift assays (EMSAs)

Oligoribonucleotides (Supplementary Table S1) were radiolabelled at their 5′ ends using T4 polynucleotide kinase (NEB, USA) and [γ-^32^P] ATP (PerkinElmer, USA). A radiolabelled 44-nt oligoribonucleotide of random sequence (t) was used in EMSAs with ssRNA. To prepare ^32^P-labelled dsRNA fragments, this random 44-nt ssRNA (t) and a 25-nt ssRNA (t), which was described previously (19), were radiolabelled and annealed to their respective and unlabelled complementary strands. In a standard EMSA reaction, a ^32^P-labelled ssRNA or dsRNA fragment was incubated with wild-type or mutant Sis10b for 30 min at 25, 37 or 55°C in 20 mM HEPES–NaOH, pH 7.0, 1 mM DTT, 100 μg/ml BSA and 6% (w/v) glycerol. These reaction mixtures were subjected to electrophoresis at a temperature identical to that of the binding reaction on a 12% or 15% non-denaturing polyacrylamide gel using 0.5× TBE. These gels were then exposed to a storage phosphor-imaging screen and analysed using ImageQuant. Dose-response curves were created by plotting the fraction of bound RNA versus indicated protein concentrations, and the apparent dissociation constant (*K*_D_) was calculated as the concentration of the protein at which half of the input RNA was retarded on the gel.

### Chemical cross-linking

Wild-type or mutant Sis10b protein (10 μg) was cross-linked with 0.5 mM dithiobis [succinimidyl propionate] (DSP, Pierce) in the presence or absence of a 25-bp dsRNA (5 μg) for 30 min at 25 or 55°C in 20 mM potassium phosphate buffer, pH 7.6, and 50 mM KCl in a total volume of 20 μl. Cross-linking reactions were stopped with the addition of 5 μl of 1.5 M Tris–HCl, pH 7.6. Samples were then subjected to 15% SDS-polyacrylamide gel electrophoresis (SDS-PAGE), and these gels were stained with Coomassie brilliant blue G-250.

### Transcription anti-termination assays in *E. coli*

To determine the nucleic acid melting activity of Sis10b in a heterologous host, wild-type *sis10b* was cloned into the NdeI and BamHI sites of the expression plasmid pINIII (28). The resulting plasmid was transformed into the *E. coli* RL211 strain (29). This strain was grown to an OD_600_ of ∼1.0 in LB medium supplemented with 100 μg/ml ampicillin. Aliquots (6 μl) of the culture were spotted onto LB plates containing 100 μg/ml ampicillin and 0.2 mM IPTG with or without 30 μg/ml chloramphenicol. The plates were incubated at 37 or 42°C for 2–3 days, and the growth was monitored. *E. coli* RL211 strains with pINIII containing *cspA* or *cspE* in place of *sis10b* or with an empty pINIII were used as positive and negative controls, respectively. For quantitative analysis of chloramphenicol-resistant colonies, *E. coli* RL211 strains with pINIII-*sis10b* or pINIII-*cspA* were cultured at 37 or 42°C to an OD_600_ of ∼0.4 in LB medium containing 100 μg/ml ampicillin and 0.2 mM IPTG. These cultures were then diluted to 10^−5^, and aliquots (200 μl) of the diluted cultures were plated onto LB plates containing 100 μg/ml ampicillin and 0.2 mM IPTG with or without 30 μg/ml chloramphenicol. The number of colonies on each plate was then counted.

### RNA unwinding assays

Oligoribonucleotides MB_40_-FITC and MB_22_-FITC, labelled at the 5′ end with a FITC fluorescent group, were allowed to anneal, respectively, to oligoribonucleotides MB_40_-BHQ1 and MB_22_-BHQ1, labelled at the 3′ end with a BHQ1 quenching group, at the molecular ratio of 1:1 (Supplementary Table S1). The annealed products, used as molecular beacons in these assays, were dsRNA fragments (*T*_m_: 59°C) with or without an unpaired and unlabelled region at one end. Wild-type or mutant Sis10b (20 μM) was incubated for 30 min at indicated temperatures with an annealed dsRNA probe (0.1 μM) in 200 mM Tris–HCl, pH 7.4 and 10 mM MgCl_2_. Changes in fluorescence were measured using a fluorescence spectrophotometer with a 460 nm excitation wavelength and a 515 nm emission wavelength.

### Strand exchange assays

Oligoribonucleotide SA was ^32^P-labelled at the 5′ end and annealed to unlabelled oligonucleotide SB at a molecular ratio of 1:1 to yield a labelled dsRNA fragment with an unpaired region at one end (Supplementary Table S1) (30). A standard reaction (12 μl) containing indicated amounts of wild-type or mutant Sis10b, 400 pM ^32^P-labelled dsRNA, 20 nM unlabelled SA strand, 40 U RNase inhibitor (Ambion), 50 mM Tris–HCl, pH 7.4, 50 mg/ml BSA, 5% (w/v) glycerol, 0.05% β-mercaptoethanol and 10 mM NaCl was set up on ice. The reaction was initiated by incubation at 37°C for 30 min and stopped with the addition of an equal volume of stop solution (10% glycerol, 0.4% SDS and 20 mM EDTA). For time-course experiments, a scaled-up reaction mixture was prepared, and aliquots were taken at intervals and mixed with an equal volume of stop solution. Samples were subjected to electrophoresis using a 15% non-denaturing polyacrylamide gel. The gel was exposed to a storage phosphor-imaging screen and analysed using ImageQuant. The percentage of ^32^P-labelled strand SA released from the duplex was plotted versus time, and the apparent first-order rate constant, *k*_1_, was determined from the plot as described previously (30).

### Promoter substitution

A genome-editing plasmid for the substitution of the promoter of *sis10b* (pGE-*sis10b*) was constructed by cloning a single spacer and sequences flanking the promoter of the *sis10b* gene (left-arm+P*_aras-SD_*+right-arm) into a *Sulfolobus* CRISPR cloning vector pSe-Rp (31,32). The spacer fragment was annealed using two synthetic complementary sequences (Supplementary Table S1) and digested with BspMI. The digested spacer was then inserted into pSe-Rp at the BspMI site. The upstream sequence (left arm) and downstream sequence (right arm) were obtained by PCR using *S. islandicus* REY15A genomic DNA as a template. The annealed arabinose-inducible promoter (P*_aras-SD_*, Supplementary Table S1) was then ligated to the right arm at the NdeI site to obtain a P*_aras-SD_*+right-arm fragment. The left arm and the P*_aras-SD_*+right-arm sequences were fused using SOE-PCR and digested with SalI and NotI. The fusion fragment was then inserted into the pSe-Rp plasmid harbouring the spacer sequence at the SalI and NotI sites to obtain plasmid pGE-*sis10b*. Plasmid pGE-*sis10b* was then transformed into *S. islandicus* E234 (33) by electroporation. The transformed cells were grown on an ACVy solid plate (34), which contained D-arabinose as the carbon source, and were screened by PCR amplification using flanking and internal primers (Supplementary Table S1). The PCR products were identified by agarose gel electrophoresis and DNA sequencing. Colony picking was repeated to obtain a pure mutant strain. The mutant strain was grown on ACVy solid plates containing 50 μg/ml 5′-fluorooritic acid (5′FOA) and 20 μg/ml uracil to eliminate the plasmid. The colonies were grown in liquid ACVy medium containing 50 μg/ml 5′FOA and 20 μg/ml uracil to yield the desired mutant strain, denoted as strain NZ1.

### Construction of complementary strains

Genes encoding wild-type Sis10b and ΔN12 were amplified by PCR using genomic DNA from *S. islandicus* REY15A as a template and cloned into the NdeI and NotI sites of the expression plasmid pSeSD under the control of the P*_araS-SD_* promoter (34). The resulting pSeSD-*sis10b* was used as a template for site-directed mutagenesis to generate the following point mutations in Sis10b: K16A, K17A, K17R, K16N/R44I, K16N/R83T and R44I/R83T (Supplementary Table S1). Genes encoding K16N/R44I/R83T and M20E/L24E/L27E/F60E were synthesized at TsingKe Biotech and cloned into the NdeI and NotI sites of pSeSD. The wild-type and mutant *sis10b* sequences were verified by DNA sequencing. The constructed expression plasmids were then transformed into strain NZ1 by electroporation, and the transformants were screened as described above except that the plasmids were maintained in these strains through growth in the absence of 5′FOA and uracil.

### Immunoblotting

Rabbit antiserum against Sis10b was generated at the Institute of Genetics and Developmental Biology, Chinese Academy of Sciences, Beijing, China. The antibody was purified first by chromatography on Protein A-Sepharose and then by affinity chromatography with resin immobilized with recombinant Sis10b. Cells from the parental and mutant *S. islandicus* strains grown in ACVy medium at 75°C were harvested at an OD_600_ of ∼0.2 and resuspended to the same cell density in PBS (137 mM NaCl, 2.7 mM KCl, 10 mM Na_2_HPO_4_, 2 mM KH_2_PO_4_, pH 7.2). An equal aliquot of each sample was then loaded onto 15% SDS-PAGE gel. After electrophoresis, proteins in the gel were electrophoretically transferred onto a PVDF membrane. The membrane was incubated successively with the antibody against Sis10b and a mouse anti-rabbit antibody conjugated with HRP. Proteins were detected by adding Enhanced Chemiluminescence (ECL) Western blot substrate and visualized with a Tanon 5200 Multi Chemiluminescent System. The intensities of the protein bands were determined using Quantity One software.

## RESULTS

### RNA binding and oligomerization by wild-type and mutant Sis10b

**Figure 1.**
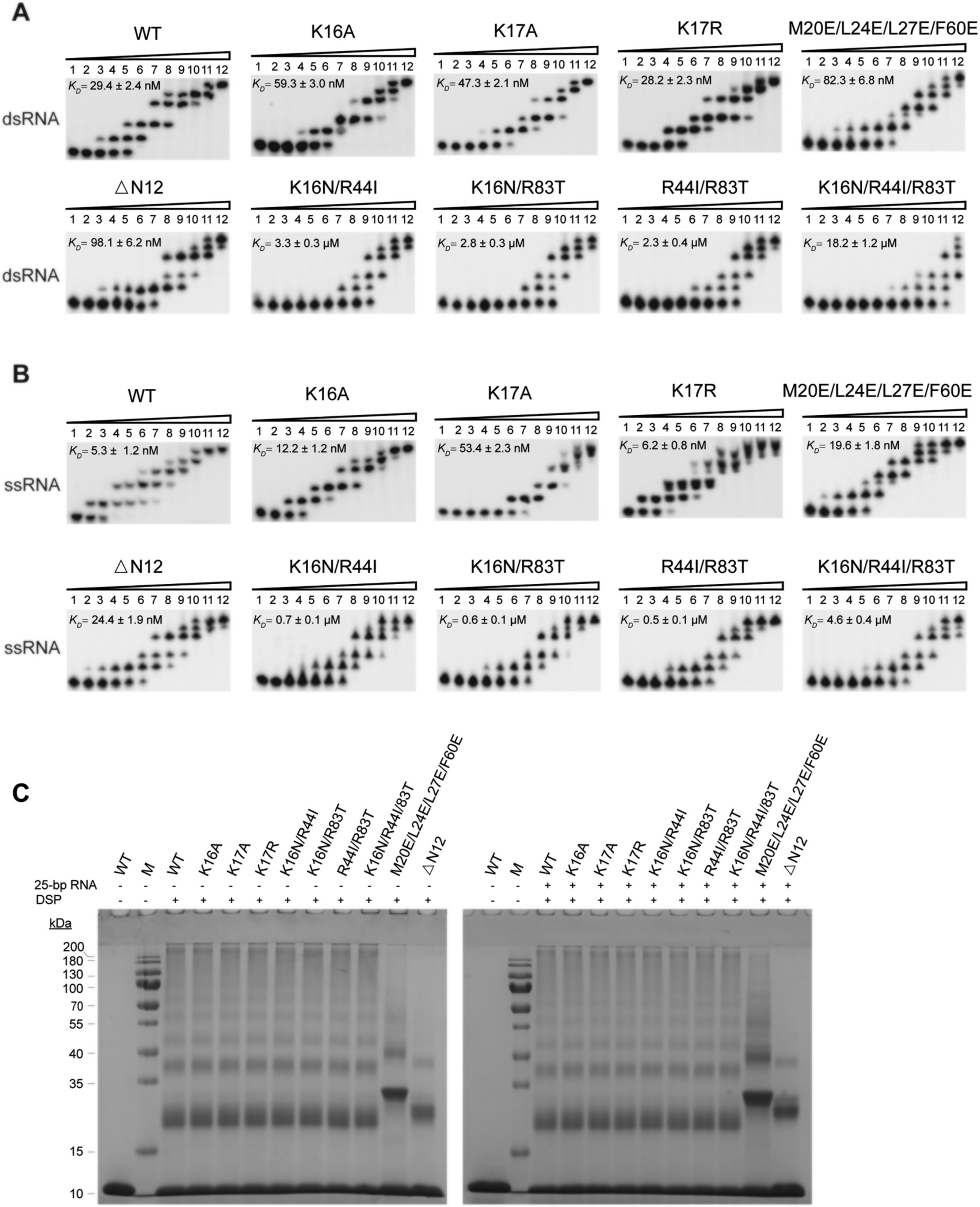
RNA binding and oligomerization by wild-type and mutant Sis10b proteins. (**A** and **B**) Analysis of the binding of wild-type and mutant Sis10b to dsRNA and ssRNA by EMSA. Wild-type or mutant Sis10b was incubated with a ^32^P-labeled 44-bp dsRNA fragment (A) or 44-nt RNA fragment (B) for 30 min at 55°C. Samples were subjected to electrophoresis at 55°C in a 12% non-denaturing polyacrylamide gel. The gels were exposed to a storage phosphor-imaging screen. For wild-type Sis10b, K16A, K17A, K17R, M20E/L24E/L27E/F60E and ΔN12, protein concentrations in lanes 1–12 were 0, 0.005, 0.01, 0.02, 0.04, 0.08, 0.16, 0.32, 0.64, 1.25, 2.5 and 5 μM, respectively. For K16N/R44I, K16N/R83T and R44I/R83T, protein concentrations in lanes 1–12 were 0, 0.04, 0.08, 0.16, 0.32, 0.64, 1.25, 2.5, 5, 10, 20 and 40 μM, respectively. For K16N/R44I/R83T, protein concentrations in lanes 1–12 were 0, 0.16, 0.32, 0.64, 1.25, 2.5, 5, 10, 15, 20, 30 and 40 μM, respectively. Apparent *K*_D_ values represent an average of three independent measurements. (**C**) Analysis of the ability of wild-type and mutant Sis10b to form oligomers. Wild-type or mutant Sis10b was cross-linked alone (left panel) or in the presence of a 25-bp dsRNA (right panel) with 0.5 mM DSP for 30 min at 55°C. Samples were analysed by 15% SDS-PAGE. Molecular mass standards are indicated.

Biochemical and structural biological studies identified several, mostly conserved, amino acid residues (corresponding to K16, K17, Y22, R42, R44, R83 etc., in Sis10b) that were involved in nucleic acid binding in the proteins of the Sac10b family (4,19,35,36) (Supplementary Figure S1B and S2). K16 is also frequently methylated (37). To learn if and how mutations at these sites would affect the cell growth, we first prepared Sis10b mutant proteins containing single, double or triple mutations at these sites (i.e. K16A, K17A, K17R, K16N/R44I, K16N/R83T, R44I/R83T, K16N/R44I/R83T, Supplementary Figure S3) and tested their ability to bind a 44-bp random dsRNA fragment (*T*_m_: 72°C) by EMSA at 55°C (Figure 1A and Supplementary Figure S4), the highest temperature at which electrophoresis could be carried out with confidence in our laboratory. When increasing amounts of wild-type Sis10b were allowed to bind the dsRNA, five shifts, representing distinct protein–RNA complexes, were generated. Among the single-point mutant proteins, K16A and K17A were ∼2.0- and 1.6-fold lower in apparent binding affinity for dsRNA (with *K*_D_ values of ∼59.3 and ∼47.3 nM, respectively) than that of wild-type Sis10b (*K*_D_ ≈ 29.4 nM), whereas K17R (*K*_D_ ≈ 28.2 nM) bound to the dsRNA as well as the wild-type protein (Figure 1A). The proteins with double-point mutations (i.e. K16N/R44I, K16N/R83T and R44I/R83T) showed a decrease in dsRNA binding affinity by ∼80- to 110-fold as compared to wild-type Sis10b. The triple-point mutant protein K16N/R44I/R83T was hardly able to bind the dsRNA even at a protein concentration of 10 μM. Similar RNA binding affinities were obtained for the wild-type and mutant proteins at 37 and 25°C (Supplementary Figures S5 and S6). These results are in general agreement with the findings in previous studies on the nucleic acid binding of *Sulfolobus* homologs of Sis10b (19,36).

We also tested the ability of wild-type and mutant Sis10b proteins to bind a 44-nt ssRNA, which was one of the strands in the 44-bp dsRNA fragment, by EMSA at 55°C (Figure 1B and Supplementary Figure S4). We found that wild-type Sis10b showed a greater affinity (∼6-fold) for ssRNA (*K*_D_ ≈ 5.3 nM) compared to dsRNA (*K*_D_ ≈ 29.4 nM) at 55°C. In addition, all mutant proteins except for K17A exhibited a much higher (∼4- to 5-fold) binding affinity for ssRNA than for dsRNA at 55°C. Strikingly, however, no similar difference in the binding affinity of K17A for the ssRNA and the dsRNA was observed at 55°C. As a comparison, we then examined the ability of wild-type Sis10b, K16A, K17A and K17R to bind the dsRNA and the ssRNA at 37°C (Supplementary Figure S5). Interestingly, although all of these proteins bound slightly better to the dsRNA than to the ssRNA (∼1.1- to 1.7-fold), none of them showed a binding preference for ssRNA over dsRNA. These results indicate that Sis10b binds to ssRNA more tightly than to dsRNA at high temperature (i.e. 55°C), and K17 is responsible for the preferential binding of this protein to ssRNA.

Proteins of the Sac10b family exist as a dimer in solution (3–5,38). Structural analysis of an Ssh10b/dsRNA complex revealed the presence of two dimer-dimer interfaces (Supplementary Figure 1A and C–F) (19). On the one hand, two adjacent Ssh10b dimers bound to the dsRNA fragment in successive major grooves to form a close intermolecular β-sheet consisting of two parallel β-strands, i.e. β1 from a monomer in one dimer and β3 from a monomer in another dimer. This intermolecular β-sheet allows the two neighbouring Ssh10b dimers to assemble into a tetramer and bind dsRNA cooperatively (19). On the other hand, two Ssh10b dimers interact through a highly conserved hydrophobic patch (Phe-60, Met-20, Leu24 and Leu-27). This interaction is probably involved in the higher-order oligomerization of Ssh10b and may help bring RNA strands together (19). It has been suggested that both types of dimer-dimer interactions are involved in oligomerization by the Sac10b family proteins (7,19,20,23,35,39). To determine if dimer-dimer interactions affected Sis10b oligomerization, we prepared an N-terminal deletion mutant protein (ΔN12), which lacked the β1 strand, and a quadruple-point mutant protein (M20E/L24E/L27E/F60E), in which the hydrophobic patch was disrupted (Supplementary Figure S3). These two mutant proteins, as well as wild-type Sis10b and the single-, double- and triple-point mutant proteins described above, were cross-linked with dithiobis [succinimidyl propionate] (DSP) in the presence or absence of a 25-bp dsRNA at either 25 or 55°C (Figure 1C and Supplementary Figure S7). The patterns of cross-linking products were similar in the presence or absence of RNA at the two temperatures, suggesting that oligomerization by wild-type and mutant Sis10b was insensitive to changes in temperature within the tested range. As expected, wild-type Sis10b, as well as all of the tested mutant proteins except for M20E/L24E/L27E/F60E and ΔN12, exhibited an identical cross-linking pattern. By comparison, both M20E/L24E/L27E/F60E and ΔN12 were considerably less efficiently cross-linked. The cross-linking efficiency of the two mutant proteins was only slightly enhanced in the presence of the RNA at a protein/RNA mass ratio of 2:1, which would allow the RNA to be completely bound by these proteins. It was noticeable that the cross-linking pattern of M20E/L24E/L27E/F60E was drastically altered. This mutant protein appeared to be cross-linked into trimers, pentamers and other unknown forms of oligomers, and no dimers or tetramers were observed, suggesting that this mutant protein differed from wild-type Sis10b in terms of protein–protein interaction. However, only a small amount of the input ΔN12 was cross-linked, and the products were mainly dimers and tetramers. EMSA assays showed that the binding affinities of ΔN12 (*K*_D_ ≈ 98.1 nM) and M20E/L24E/L27E/F60E (*K*_D_ ≈ 82.3 nM) for dsRNA were lower than that of the wild-type protein by ∼3.3- and ∼2.8 fold, respectively (Figure 1A). Since dimer-dimer interaction through either the intermolecular β–sheet or the hydrophobic patch has been suggested to be involved in cooperative nucleic acid binding by the Sac10b family proteins (7,19,35,39), we speculated that the observed decrease in the affinity of these mutant proteins for dsRNA could be attributed to changes in the RNA binding behaviour of these proteins. We also noticed that the two mutant proteins were less capable of generating the second shift, presumably formed by the binding of two Sis10b dimers to the RNA, and M20E/L24E/L27E/F60E formed one more shift than the wild-type protein. The altered gel shift pattern of these mutant proteins remains to be understood but it was likely related to the defects in protein-protein interaction. We concluded that the mutant Sis10b proteins defective in either of the two forms of dimer-dimer interaction had a significantly reduced ability to form oligomers but only a moderately decreased ability to bind dsRNA.

### Sis10b exhibits RNA chaperone activity

**Figure 2.**
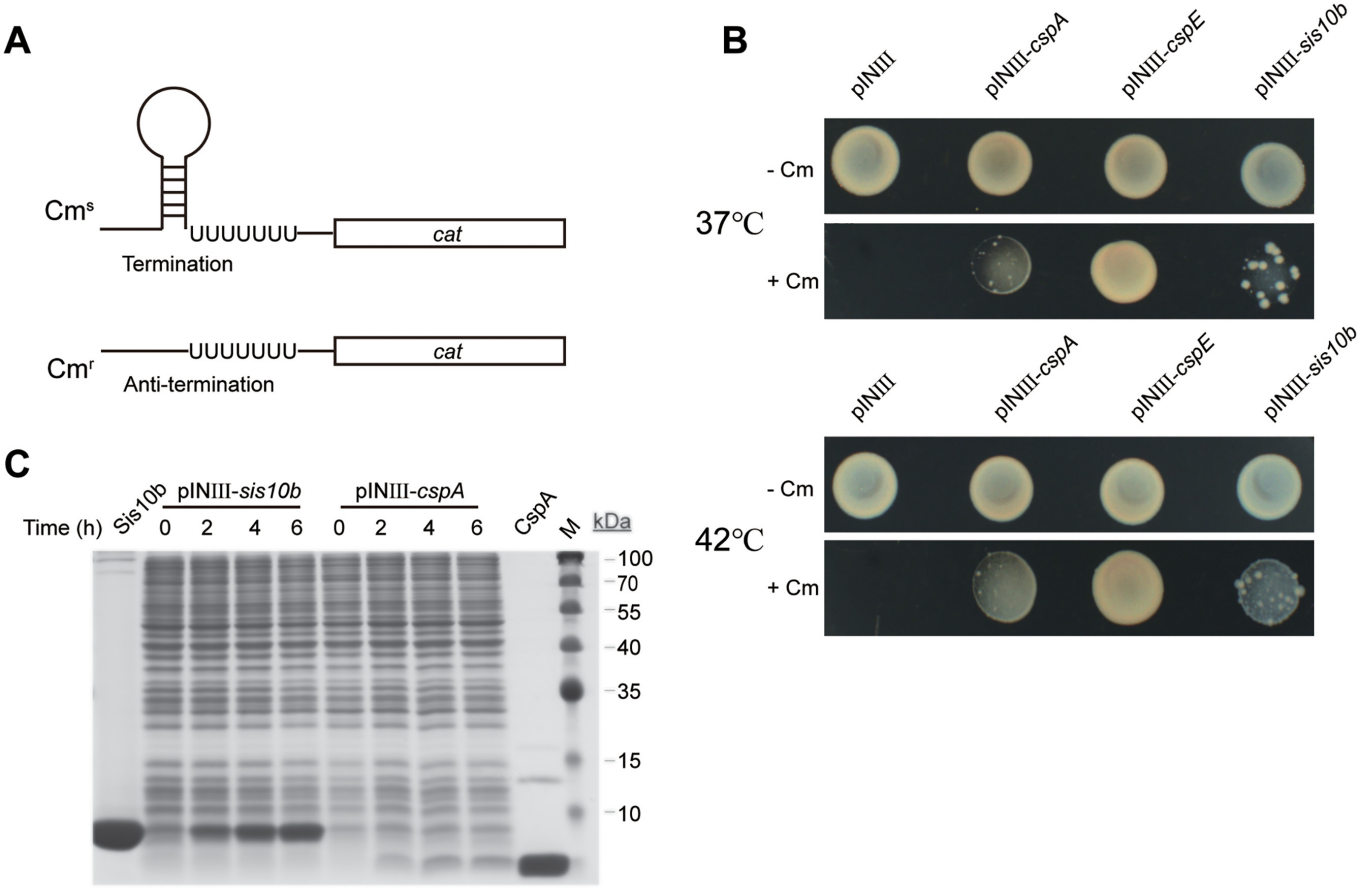
Transcription anti-termination assays in *E. coli* RL211. (**A**) A diagram showing a chloramphenicol acetyl transferase *cat* gene cassette (chloramphenicol resistance) located downstream of *trpL* terminators as a reporter in *E. coli* RL211. (**B**) *E. coli* RL211 cells transformed with an empty pINIII vector (ampicillin resistance) alone or pINIII carrying *cspA*, *cspE* or *sis10b* were cultured and adjusted to an OD_600_ of 1.0 by dilution with fresh medium, and an aliquot of each diluted cell suspension was plated onto a LB plate containing 100 μg/ml ampicillin and 0.2 mM IPTG with or without 30 μg/ml chloramphenicol (+/−Cm). The plates were incubated at 37 or 42°C for 2–3 days. (**C**) Analysis of total cellular proteins by SDS-PAGE. Purified Sis10b and CspA as well as the time after IPTG induction are indicated.

It was previously shown by RNase T1 footprinting that binding by Ssh10b destabilized base pairing in the secondary structure of RNA (19). This raised the possibility that the Sac10b family proteins may function as an RNA chaperone. To test this possibility, we first employed an assay involving the use of an *E. coli* RL211 system, which carries *cat*, a gene encoding the chloramphenicol acetyl transferase (chloramphenicol resistance) cassette, located downstream of a strong *trpL* terminator as a reporter gene (29) (Figure 2A). If a test protein produced from a plasmid introduced into the strain exhibits RNA chaperone activity, the hairpin structure of the *trpL* terminator was unwound, and the downstream *cat* gene was expressed. As a result, the *E. coli* strain would become resistant to chloramphenicol (28,40,41). As shown in Figure 2B, like the known RNA chaperones CspA and CspE from *E. coli* (28,42,43), plasmid-encoded Sis10b significantly enhanced the chloramphenicol resistance of the RL211 strain at 37°C. Therefore, Sis10b appeared to be able to unwind this hairpin structure, allowing the expression of the downstream *cat* gene in an *E. coli* anti-termination assay system. Since the cellular level of Sis10b was 4–5-fold higher than that of CspA, as estimated by SDS-PAGE (Figure 2C), and the strain producing Sis10b was significantly less resistant to chloramphenicol than the strain producing CspA, as the fractions of the chloramphenicol-resistant colonies among the total colonies were 11% and 35% for the former and the latter, respectively (Figure 2B and Supplementary Table S2), Sis10b appeared to have a much lower chaperone activity than CspA at 37°C. Given the hyperthermal origin of Sis10b, we then tested the effect of temperature on the chaperone activity of these proteins by incubating these strains at 42°C. The strain producing Sis10b showed similar chloramphenicol-resistant growth to that producing CspA, with 34% and 45% of the colonies, for the former and the latter, respectively, being resistant to chloramphenicol (Figure 2B and Supplementary Table S2), raising the possibility that Sis10b might exhibit higher chaperone activity at higher temperatures.

**Figure 3.**
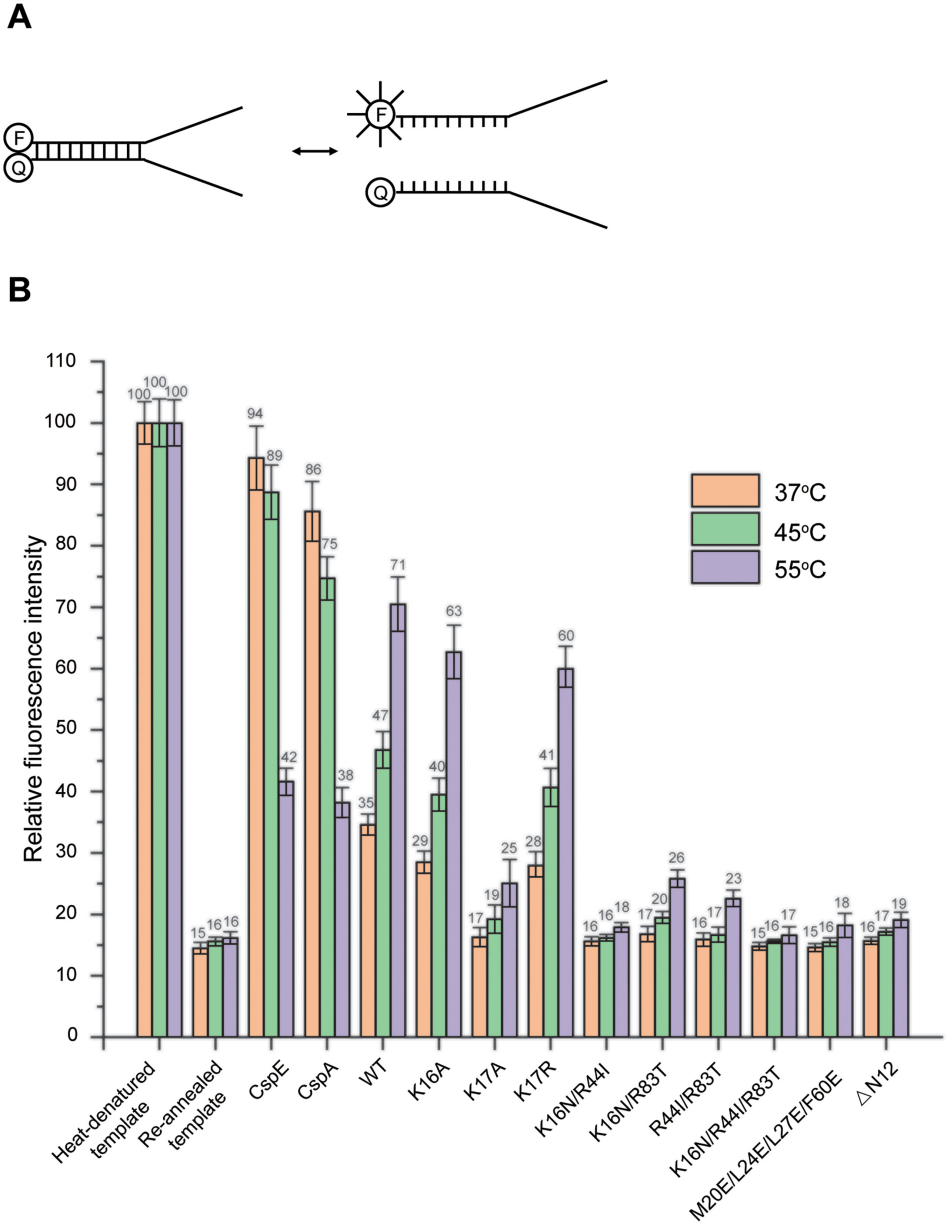
Unwinding of dsRNA by wild-type and mutant Sis10b. (**A**) A sketch of the FITC-labeled partially complementary dsRNA template used in unwinding assays. (**B**) The dsRNA unwinding activities of wild-type and mutant Sis10b. Each protein (20 μM) was mixed with the FITC-labelled template (0.1 μM), and the change in fluorescence was monitored at indicated temperatures. The fluorescence of the heat-denatured, FITC-labeled template was defined as 100% and that of the annealed dsRNA template was a control. Each number represents an average of three independent measurements.

Since an RNA chaperone is generally capable of unwinding dsRNA, we further examined the RNA chaperone activity of Sis10b and its temperature dependence using a molecular beacon assay (MB). Two partially complementary 40-nt ssRNA strands were prepared and annealed (*T*_m_: 59°C; Figure 3A). One of the strands was labelled with FITC at the 5′ end, and the other strand was labeled with a fluorescence quencher (BHQ1) at the 3′ end. The fluorescence of the partial duplex RNA was ∼15% of that of the heat-denatured ssRNA strands due to the quenching of the FITC fluorescence by BHQ1. The addition of wild-type Sis10b raised the fluorescence intensity to 35% at 37°C (Figure 3B). A further increase in temperature to 55°C was accompanied by an increase in fluorescence to 71%. In comparison, while CspE and CspA were more efficient than Sis10b in RNA unwinding (94% and 86%, respectively) at 37°C, in agreement with the observation in the above *in vivo* assay, their unwinding activity decreased drastically to 42% and 38%, respectively, at 55°C, presumably because of the partial denaturation and decrease in the activity of these *E. coli* proteins at a higher temperature (44) (Figure 3B). We also determined the unwinding activity of the Sis10b mutant proteins. Both K16A and K17R were similar to wild-type Sis10b in RNA unwinding. Interestingly, K17A was inactive in unwinding (17%) at 37°C and barely active (25%) at 55°C, suggesting that a basic residue at the 17th position of the protein may play an important role in unwinding activity. This was consistent with the finding that K17 was a highly conserved residue among the Sac10b protein family members with a few exceptions in which it was replaced by arginine (Supplementary Figure S2). However, K16, an amino acid residue involved in RNA binding, was not essential for the unwinding activity of the protein. Other mutants, such as K16N/R44I, K16N/R83T, R44I/R83T, K16N/R44I/R83T, ΔN12 and M20E/L24E/L27E/F60E, appeared to have lost unwinding activity, as anticipated from their defects in nucleic acid binding or oligomerization.

To test if the single-stranded fork region in the template affected the unwinding activity of Sis10b, we conducted MB assays on a template comprising only the complementary portion of the template used in the above assay (Supplementary Figure S8A and Supplementary Table S1). We found that Sis10b, as well as CspA and CspE, had lower RNA unwinding activity on the blunt-ended template than on a forked template (Supplementary Figure S8B). Therefore, the single-stranded region in the forked template facilitated RNA unwinding by Sis10b.

**Figure 4.**
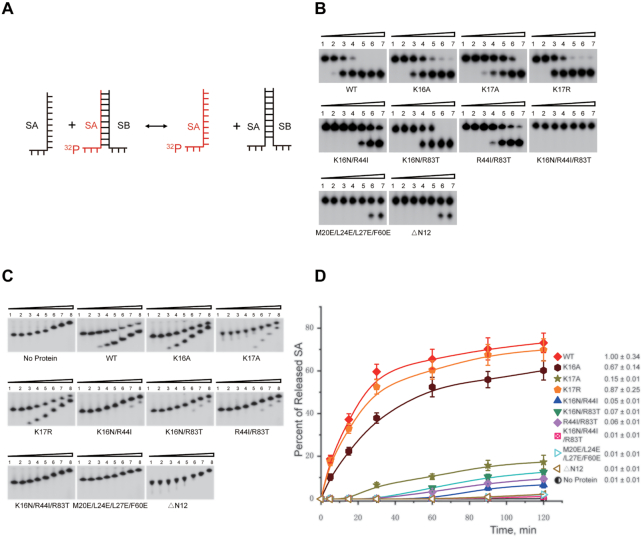
RNA strand exchange by wild-type and mutant Sis10b. (**A**) Schematic diagram of the templates in RNA strand exchange assays. A partially complementary RNA duplex was ^32^P-labelled on strand SA. (**B**) RNA strand exchange by wild-type and mutant Sis10b. Wild-type or mutant Sis10b was incubated with the ^32^P-labelled duplex template in the presence of a 50-fold excess of unlabelled SA for 30 min at 37°C. Reactions were stopped, and samples were subjected to electrophoresis in a 15% non-denaturing polyacrylamide gel. The gels were exposed to a storage phosphor-imaging screen. Protein concentrations in lanes 1–7 were 0, 0.25, 1, 4, 15, 60 and 120 μM, respectively. (**C**) The time course of strand exchange by wild-type and mutant Sis10b. The ^32^P-labelled duplex RNA was incubated with 1 μM wild-type Sis10b or indicated mutant Sis10b protein in the presence of a 50-fold excess of unlabelled SA. Samples were taken at 0, 1, 5, 15, 30, 60, 90 and 120 min, and the reaction was stopped. The samples were loaded on a running 15% non-denaturing polyacrylamide gel. (**D**) Quantification of the time course data from (C). The fraction of ^32^P-labelled SA strand released from the duplex RNA substrate was plotted against time, and the apparent first-order rate constant (*k*_1_) was determined from the plot (30). The relative rates of strand exchange as a fraction of wild-type Sis10b are shown. For wild-type Sis10b, *k*_1_ = (1.15 ± 0.40) x 10^−2^/s. Each value is an average of three independent measurements.

To further verify the RNA chaperone activity of Sis10b, we performed an RNA strand exchange assay which involved the use of an RNA duplex (*T*_m_: 43°C) with one of the strands (SA) labelled with ^32^P. In the presence of unlabelled SA at a 50-fold molar excess over the labeled SA, strand exchange mediated by wild-type or mutant Sis10b at 37°C was monitored by native gel electrophoresis over a 2-h period (Figure 4A). As shown in Figure 4B, wild-type Sis10b was most efficient in strand exchange. The rate constants (*k*_1_) of strand exchange by K17R and K16A were slightly lower (by ∼1.2- and 1.4-fold, respectively) than that of the wild-type protein (Figure 4C and D). However, the rate constant of the reaction catalysed by K17A was significantly lower than that of wild-type Sis10b (∼6.6-fold), pointing again to the key role of a basic residue at the 17th position of the protein in RNA chaperone activity. K16N/R44I, K16N/R83T and R44I/R83T were inefficient, whereas K16N/R44I/R83T, M20E/L24E/L27E/F60E and ΔN12 were nearly inactive in catalysing strand exchange.

Taken together, our data indicate that Sis10b is capable of RNA unfolding/unwinding and strand exchange and is thus an RNA chaperone. Furthermore, a basic amino acid residue at position 17 is essential for the chaperone activity of Sis10b.

### Effect of Sis10b mutations on the growth of *S. islandicus*

**Figure 5.**
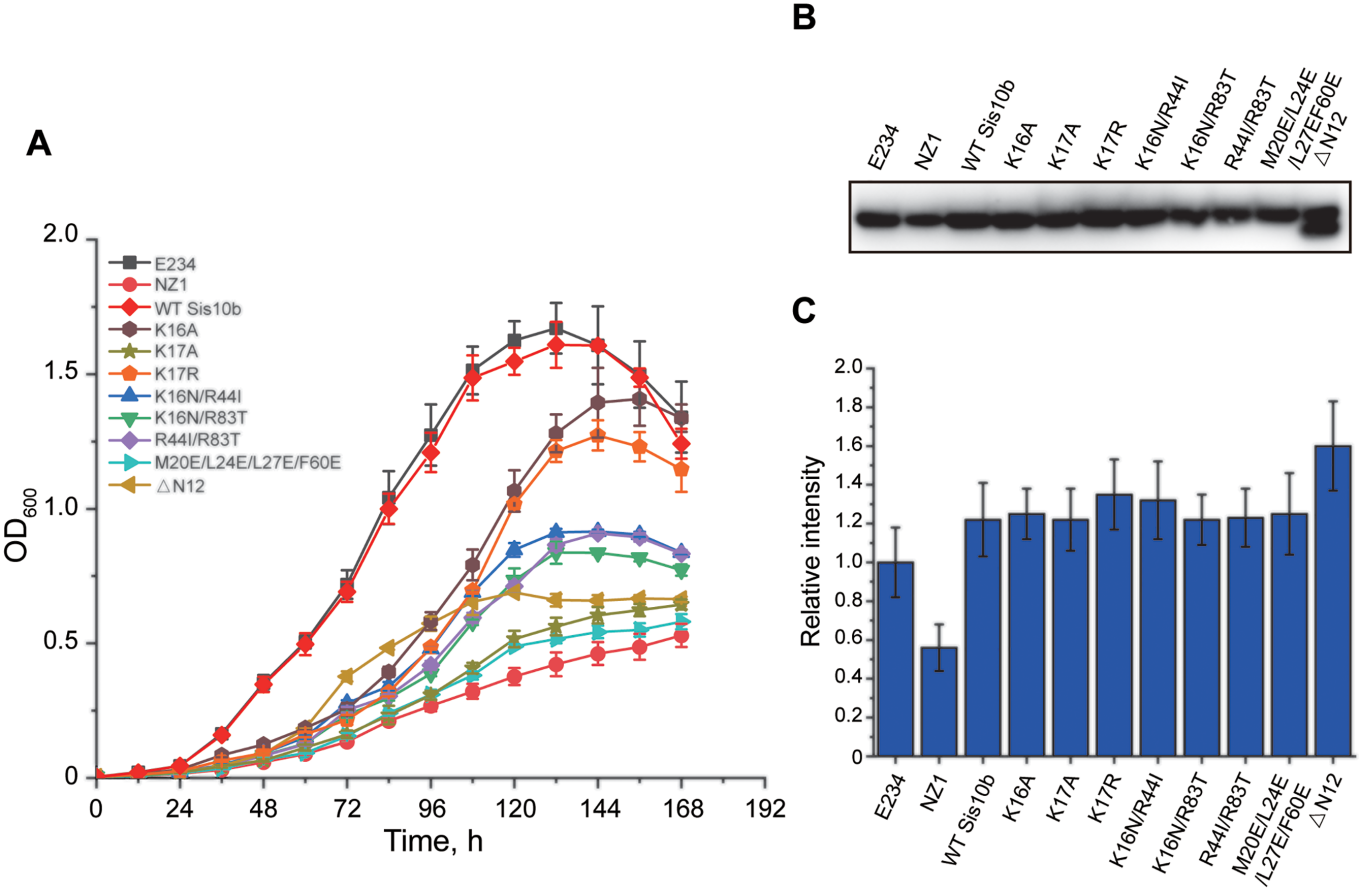
Effect of the expression of plasmid-encoded wild-type and mutant Sis10b on the growth of the Sis10b knockdown mutant strain NZ1. (**A**) Growth curves of NZ1 complemented by plasmid-encoded wild-type and mutant Sis10b. NZ1 was transformed with plasmid encoding wild-type or mutant Sis10b. The transformants were incubated in liquid ACVy medium, and the OD_600_ values were recorded at indicated times. Each growth curve was determined in triplicate. (**B**) Analysis of the cellular Sis10b contents in NZ1 and complementary strains. The strains were grown to the early-exponential phase, and total cellular proteins were resolved by SDS-PAGE. Sis10b in each sample was determined by immunoblotting with an antibody against Sis10b. (**C**) Quantitative comparison of the cellular levels of wild-type and mutant Sis10b in NZ1 and complementary strains. The intensities of the Sis10b bands, detected above by immunoblotting, were quantified using Quantity One software. Each number is an average of three independent measurements.

Finally, we sought to determine the physiological consequence of introducing the above mutations, especially the mutations at K17, into Sis10b. Since Sis10b is essential for the growth of the organism, we constructed a *sis10b* knockdown mutant in *S. islandicus* by replacing the native promoter of *sis10b* in the genome with an arabinose-inducible promoter (P*_aras-SD_*) (34) using CRISPR-based genome editing (31). The knockdown strain, denoted NZ1, was unable to grow in the absence of arabinose but, when induced by the addition of arabinose, attained a slow growth rate, with the OD_600_ peaking at ∼0.4, compared to a maximum OD_600_ of ∼1.7 for the parental strain under the same growth conditions (Figure 5A). The cellular level of Sis10b in the mutant was about half of that in the parental strain (Figure 5B and C). We then prepared a complementary strain by introducing a pSeSD-derived expression plasmid carrying wild-type *sis10b* under the control of the P*_aras-SD_* promoter into the mutant strain. Growth was restored in the complementary strain, which contained slightly more Sis10b than the parental strain (∼1.2-fold), when grown in the presence of arabinose (Figure 5). Clearly, a minimum level of Sis10b in the cell was required for the survival of the organism. The availability of the *sis10b* knockdown mutant NZ1 permitted the analysis of the *in vivo* function of the Sis10b mutations by complementation.

Genes encoding the mutant proteins described above were cloned into plasmid pSeSD under the control of the P*_aras-SD_* promoter, and each plasmid was introduced into NZ1. The growth of the resulting transformants in the presence of arabinose was monitored, and the cellular level of total Sis10b in each transformant was determined (Figure 5). All of the transformants, except for that transformed with the plasmid encoding K16N/R44I/R83T, were obtained. The cellular levels of total Sis10b in the transformants producing K16A, K17A, K17R, K16N/R44I, K16N/R83T, R44I/R83T and M20E/L24E/L27E/F60E were 1.2–1.3 times as high as that of the parental strain (Figure 5B and C). Total Sis10b in the transformant overproducing ΔN12 was 1.6–1.8 times as much as that of the parental strain (Figure 5B and C), presumably because the lack of N-terminal acetylation led to an increase in the plasmid-encoded mutant protein (37). As shown in Figure 5A, the growth of NZ1 was most effectively restored by the overproduction of K16A or K17R among the tested mutant proteins. K16N/R44I, K16N/R83T, R44I/R83T and ΔN12 were less effective in restoring the growth of the strain. M20E/L24E/L27E/F60E was nearly inactive in our complementation assay. These results indicate that nucleic-acid binding and oligomerization are both required for Sis10b to serve its physiological function. Notably, overproduction of K17A was barely able to restore the growth of NZ1. Therefore, a basic residue at the 17th position is essential for the *in vivo* role of the protein.

## DISCUSSION

*Sulfolobus* members of the Sac10b family have long been regarded as chromatin proteins (3,5–7,23,45,46). However, several lines of evidence suggest that these proteins are bound to RNA and presumably involved in RNA metabolism *in vivo* (1,8,15,16,19,47). In this report, we demonstrate for the first time that Sis10b is an RNA chaperone. The protein unfolded an RNA hairpin, when overproduced in a heterologous host, unwound dsRNA and promoted RNA strand exchange without consuming ATP *in vitro*. These results, as well as the previous findings that the protein binds RNA without apparent sequence specificity and is capable of destabilizing RNA secondary structures (19), are all consistent with this notion. Proteins of the Sac10b family are known to exist in abundance in thermophilic and hyperthermophilic archaea, as exemplified by the estimate that Ssh10b accounts for ∼2% of total cell proteins (8,48). Binding by the Sac10b proteins may serve to protect RNAs from thermal stress. At the same time, the chaperone activity of these proteins may permit the use or processing of RNAs by specific cellular machinery. It was shown previously that archaeal proteins of the Sac10b family were enriched at ribosomes (5,8) and RNase P (9,49). Sis10b was also found to be associated with exosomes through its interaction with RNA bound by multiprotein complexes (Supplementary Figure S9). These observations raise the possibility that the Sac10b proteins can facilitate processes such as translation and RNA degradation, which presumably require the destabilization or refolding of RNA structure (19,50).

To gain more insights into the structure-function relationship, especially the mechanistic aspects of the chaperone activity, of Sis10b, we examined Sis10b containing mutations at residues involved in nucleic acid binding and protein oligomerization using both *in vitro* and *in vivo* assays. We showed that both RNA binding and protein oligomerization were required for the RNA chaperone activity of Sis10b, as expected. Dimer-dimer interactions, which apparently occurred in the oligomerization of Sis10b, would presumably serve to bring together two RNA strands or duplexes in the formation of higher-order RNA structures and in strand exchange reactions. Surprisingly, K17, the most highly conserved residue in the Sac10b family with R as the only known substitution in very few species (Supplementary Figure S2), appeared to be critically required for the chaperone activity and the physiological function of Sis10b. K17, located in the flexible loop connecting β1 and α1, is part of the nucleic acid-binding interface that also contains K16, Y22, R42 and R44. K17 was found to interact only weakly with RNA in a Ssh10b-dsRNA crystal (19) but not with DNA in an Ape10b2-dsDNA crystal (35). By comparison, the neighbouring K16 residue, interacts more strongly with both DNA and RNA in these complex crystals. In agreement with the structural findings, the affinity of K17A for dsRNA was only slightly lower (by 1.6-fold) than that of wild-type Sis10b, whereas K16A was 2.0-fold lower in dsRNA binding than the wild-type protein. In addition, K17R bound dsRNA as well as wild-type Sis10b. Neither K17A nor K16A differed from the wild-type protein in their ability to oligomerize. However, the ability of K17A to unwind dsRNA at an elevated temperature (i.e. 55°C) and to promote RNA strand change was significantly lower than either wild-type Sis10b, K16A or K17R. Therefore, K17, or a basic residue at the 17th position, appears to play an important role in the chaperone activity of Sis10b.

Preferential binding to ssRNA over dsRNA is required for an RNA chaperone to unfold RNA (51–54). Intriguingly, with an increasing temperature in the range tested, the affinity of Sis10b for dsRNA increased only slightly but that for ssRNA increased substantially, and the affinity of Sis10b for ssRNA was ∼6-fold higher than that for dsRNA at 55°C. Both K16A and K17R behaved in a similar fashion to wild-type Sis10b in preferential binding to ssRNA over dsRNA at 55°C (Figure 1A and B). However, K17A showed no difference in binding to ssRNA and dsRNA at 55°C, suggesting that K17 plays an important role in ssRNA binding, especially at high temperature. The impact of temperature on the affinity of Sis10b for the two different forms of the RNA might be attributed to the known effect of temperature on the conformation of the protein (5,6). Two forms of Ssh10b homodimers are known to co-exist in solution, and the slow *cis-trans* isomerization of the L61-P62 peptide bond is the key factor responsible for the conformational heterogeneity of a Ssh10b homodimer (6). The T-form dimer, with the L61-P62 bond in the *trans* conformation, dominates at higher temperature, whereas the population of the C-form dimer, with the bond in the *cis* conformation, increases with decreasing temperature. This was presumably consistent with the observed changes in the mode of binding to dsDNA and the ability to constrain negative DNA supercoils by Ssh10b in a temperature-dependent manner (5,6). In support of the role of the L61–P62 bond as a temperature switch for protein conformation, P62 is especially conserved in thermophilic and hyperthermophilic archaea, including those from Crenarchaeota, Euryarchaeota and Korarchaeota. Most of these organisms contain X61-P62 (X stands for L, I or M) in at least one of their Sac10b homologs (Supplementary Figure S10). Therefore, it is possible that this temperature-dependent conformational change allows Sis10b to interact more strongly with ssRNA than dsRNA, and K17 is crucially involved in the enhanced binding of the protein to ssRNA at higher temperatures.

We also determined the physiological consequences of mutating Sis10b at various specific residues using a complementation assay in a Sis10b knockdown mutant strain since the knockout mutation of this protein is lethal. During the construction of our knockdown mutant strain, we found that a minimal level of Sis10b was required for the survival of *S. islandicus*. Our mutant strain was able to sustain slow but readily detectable growth when Sis10b was present at about half of wild-type levels, as expected from the known dependence of the activity of a chaperone on molecular crowding effect (55–57). As demonstrated in complementation experiments, mutant Sis10b proteins with defects in RNA binding or oligomerization were able to restore the growth of the mutant strain to various extents, which appeared to be proportional to the remaining binding or oligomerization activity of the mutant protein in most cases. Notably, both K16A and K17R effectively restored the growth of the mutant. However, K17A was a striking exception. Overproduction of the K17A mutant protein was barely able to restore the growth in the knockdown mutant. As a further indication of the importance of K17, we were able to construct K16A and K17R mutant strains but were unsuccessful in making a K17A strain despite repeated efforts (37). Therefore, a basic residue at the 17th position of Sis10b may be required for the *in vivo* role of the protein. Further studies are underway to shed light on the role of K17 in preferential binding to ssRNA at high temperature.

## Supplementary Material

gkaa656_Supplemental_File

## Notes

Present address: Li Guo, State Key Laboratory of Electrical Insulation and Power Equipment, Center for Plasma Biomedicine, Xi'an Jiaotong University, Xi'an 710049, China.
