# Supplementary material for: The Sac10b homolog from Sulfolobus islandicus is an RNA chaperone
Source: Nucleic Acids Res. 2020 Aug 6;48(16):9273–84. doi: 10.1093/nar/gkaa656 (PMC7498313; doi:10.1093/nar/gkaa656)
Supplement: gkaa656_Supplemental_File [file gkaa656_supplemental_file.pdf]

The Sac10b homolog from *Sulfolobus islandicus* is an RNA chaperone

Ningning Zhang[1,2], Li Guo[1] and Li Huang[1,2] *

[1] State Key Laboratory of Microbial Resources, Institute of Microbiology, Chinese Academy of Sciences, No.1 Beichen West Road, Chaoyang District, Beijing 100101, China.

[2] College of Life Sciences, University of Chinese Academy of Sciences, No.19A Yuquan Road, Shijingshan District, Beijing 100049, China.

* To whom correspondence should be addressed. Tel: 86-10-64807430; Fax: 86-10-64807429; Email: huangl@sun.im.ac.cn

MATERIAL AND METHODS

Construction of a mutant *S. islandicus* strain encoding DnaG-Strep-His_{10}

An *S. islandicus* mutant strain that synthesized DnaG as a hybrid protein containing a Strep tag and a His_{10} tag in succession at its C-terminus was constructed by amplifying separately DNA fragments containing the *dnaG* gene (SiRe_1914) and the flanking regions immediately upstream and downstream of *dnaG* by PCR from the *S. islandicus* genomic DNA. A sequence encoding Strep and His_{10} was included in the upstream primer for the *dnaG* gene. The gene replacement vector was constructed by inserting a DNA fragment containing the *pyrEF* gene (SSO0615 and SSO0616), which was prepared from *S. solfotaricus* P2 by PCR, flanked by the upstream and downstream sequences of the *dnaG* gene of *S. islandicus* into plasmid pUC18. The plasmid was propagated in *E. coli*, isolated, linearized and transformed into *S. islandicus* E234. The transformed cells were plated on SCVy plates and incubated at 75°C. Colonies were transferred to SCVy liquid medium and incubated at 75°C. Successful replacement of the wild type *dnaG* gene with the gene encoding a DnaG-Strep-His_{10} hybrid was confirmed by PCR and DNA sequencing. The gene replacement strain was denoted *S. islandicus* LH1.

Isolation of DnaG-Strep-His_{10} hybrid protein and exosome complexes

S. islandicus LH1 cells were harvested by centrifugation from 2-liter cultures. The cells were resuspended in 30 ml of buffer A [20 mM Tris-Cl, pH 8.0, 150 mM NaCl, 10% (w/v) glycerol], and lysed by sonication. After centrifugation, the pellet was resuspened in buffer A and loaded onto a 1-ml HiTrap chelating column pre-equilibrated with buffer A. The column was washed with buffer A, and proteins were eluted using a linear imidazole gradient from buffer A to buffer B [20 mM Tris-Cl, pH 8.0, 150 mM NaCl, 500 mM imidazole, 10% (w/v) glycerol]. Fractions containing DnaG-Strep-His_{10} were identified by immunoblotting with anti-His_{10} or anti-Sis10b antibody, pooled and dialyzed against buffer C (20 mM Tris-Cl, pH 8.0, 150 mM NaCl, 1 mM EDTA, 1 mM dithiothreitol). To test the sensitivity of the association of Sis10b with the exosome to RNase treatment, the HiTrap column fractions (0.5 mL) containing DnaG-Strep-His_{10} were incubated for 60 min at 37°C with RNase A (50 U, Fermentas) and RNase T1 (25 U, Fermentas). The samples were loaded onto a Superdex 200 column equilibrated with buffer A [20 mM Tris-Cl, pH 8.0, 150 mM NaCl, 10% (w/v) glycerol]. The column was washed with buffer A. Eluted fractions were detected by silver staining and immunoblotting with anti-Sis10b antibody.

Supplementary Figures

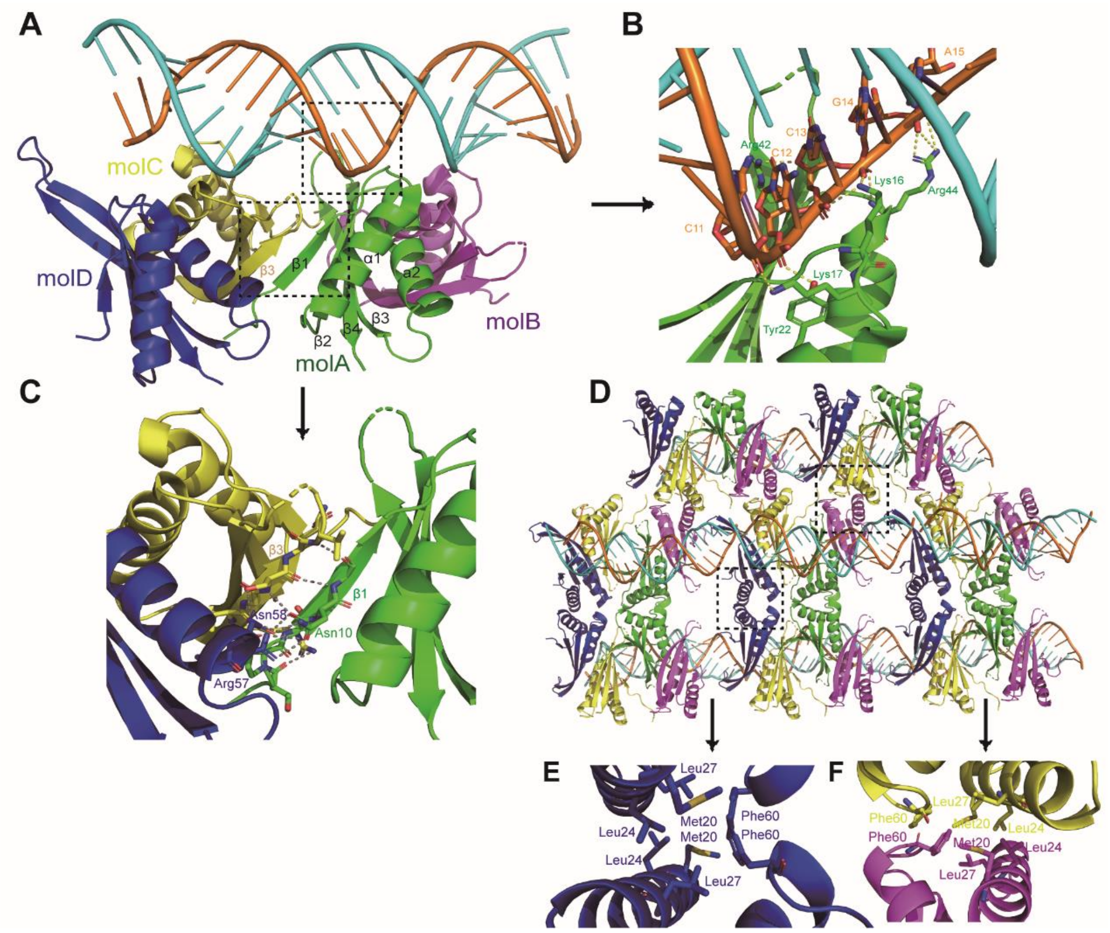

Supplementary Figure S1. A structural view of amino acid residues of Ssh10b, which are involved in RNA binding and dimer-dimer interaction. (A) Ribbon diagrams of the Ssh10b-RNA complex (PDB code: 3WBM21). Four Ssh10b monomers (A, B, C and D) are colored in green, magenta, yellow and blue, respectively. The secondary structural elements of monomer A are labelled. Two complementary RNA strands are colored in orange and cyan, respectively. (B) Interfaces between the RNA and molecule A. Interacting residues and nucleotides are labelled and shown as sticks. Hydrogen bonds are shown as dashed lines. (C) A close-up view of the intra-dsRNA fibre dimer-dimer interface between two adjacently bound Ssh10b dimers. Residues involved in the interaction between the two Ssh10b dimers are labelled and shown as sticks. (D) A ribbon diagram of Ssh10b dimers in the complex crystal. (E, F) A close-up view of the inter-dsRNA fibre dimer-dimer interactions formed through the hydrophobic patch of the interacting Ssh10b dimers in the complex crystal.

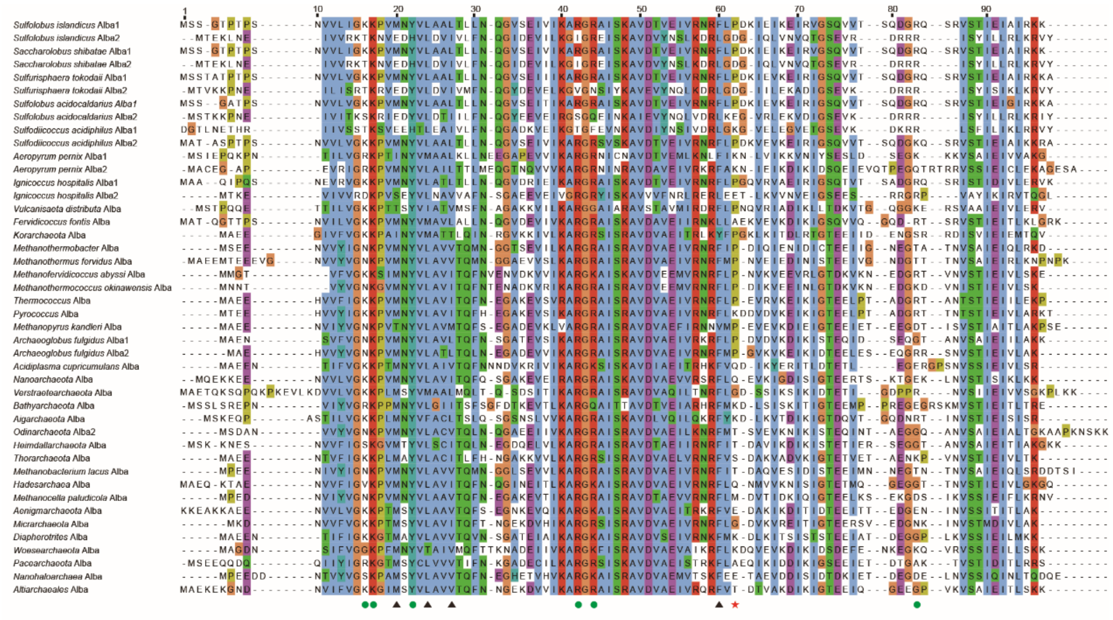

Supplementary Figure S2. A sequence alignment of members of the Sac10b family (Alba) in Archaea. Residues involved in RNA binding and dimer-dimer interaction are indicated with a green dot and a black triangle, respectively. P62 is marked with a red asterisk.

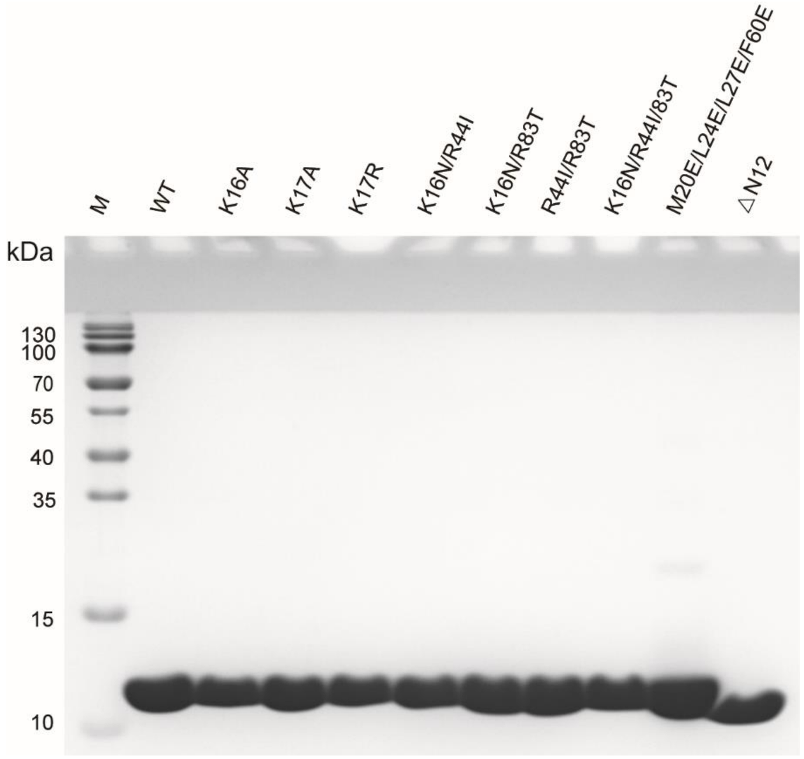

Supplementary Figure S3. Analysis of purified recombinant wild-type and mutant Sis10b proteins by SDS-PAGE. A sample (~5 μg) of each protein was subjected to 15% SDS-PAGE and the gel was stained with Coomassie Brilliant Blue G-250. Lane M, molecular mass standards.

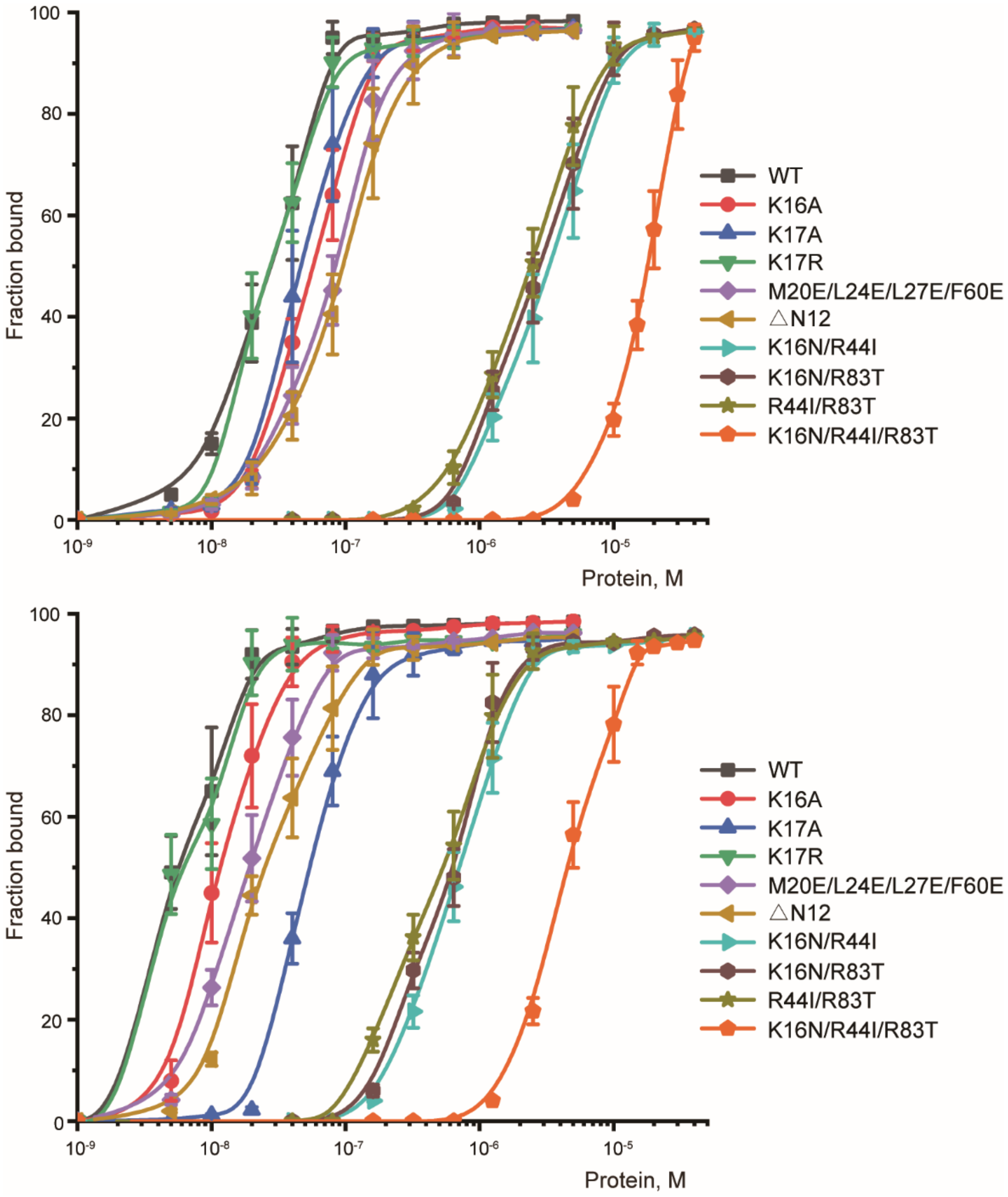

Supplementary Figure S4. Quantification of the data in the EMSA assays from Fig. 1A (up panel) and B (down panel). The fraction of the protein-bound RNA was plotted against the concentration of the protein. Apparent K_D was determined by the concentration of the protein at which half of the input RNA was retarded. Each K_D value represents an average of three independent measurements.

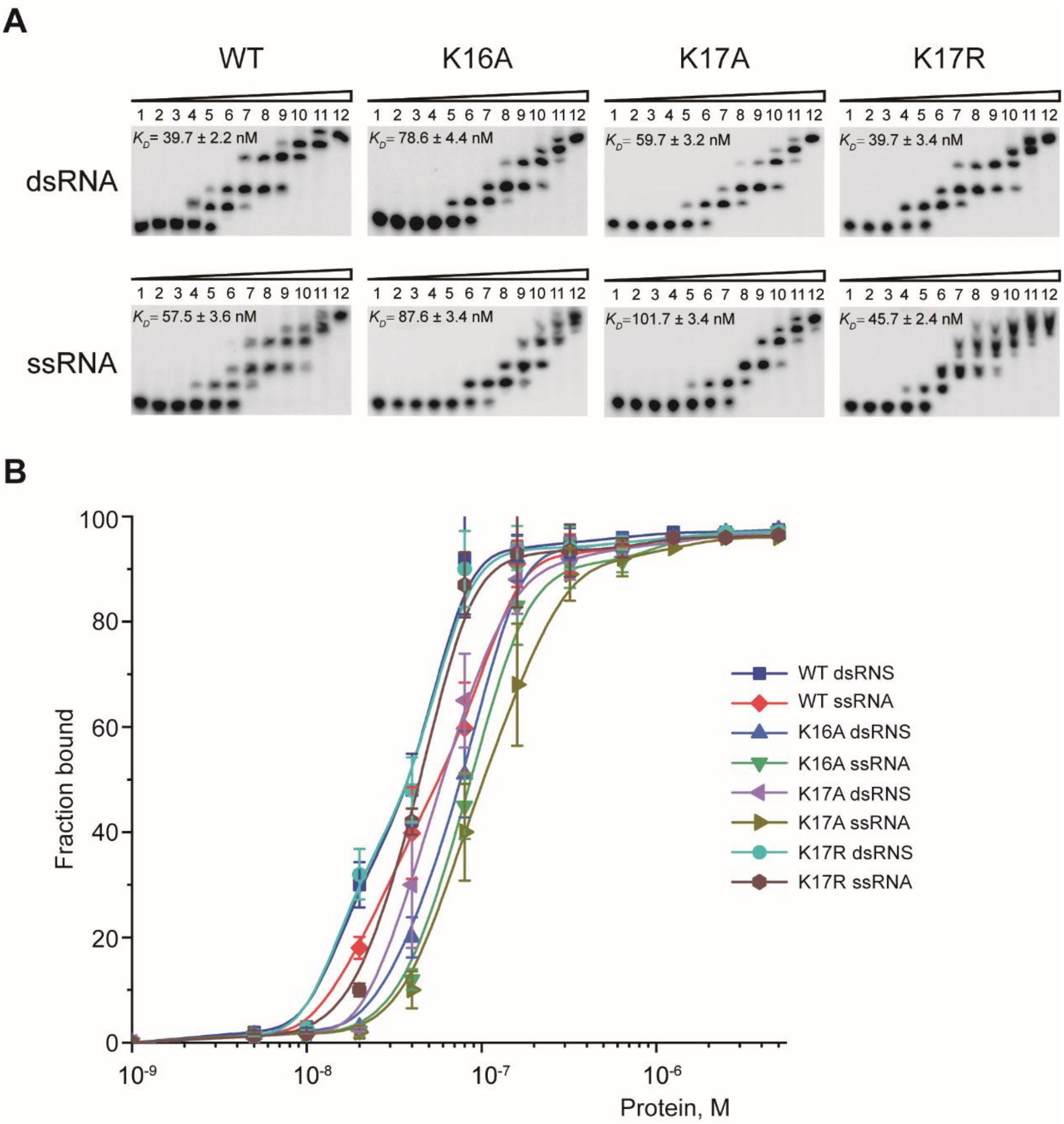

Supplementary Figure S5. Binding of wild-type and mutant Sis10b to dsRNA and ssRNA at 37°C. (A) Wild-type Sis10b, K16A, K17A or K17R was incubated with ^{32}P-labelled 44-bp dsRNA or 44-nt ssRNA for 30 min at 37°C. Samples were subjected to electrophoresis in a 12% polyacrylamide gel at 37°C. Protein concentrations in lanes 1–12 were 0, 0.005, 0.01, 0.02, 0.04, 0.08, 0.16, 0.32, 0.64, 1.25, 2.5 and 5 μM, respectively. (B) Quantification of the data in the EMSA assays. The fraction of the protein-bound RNA was plotted against the concentration of the protein. Apparent K_D was determined by the concentration of the protein at which half of the input RNA was retarded. Each K_D value represents an average of three independent measurements.

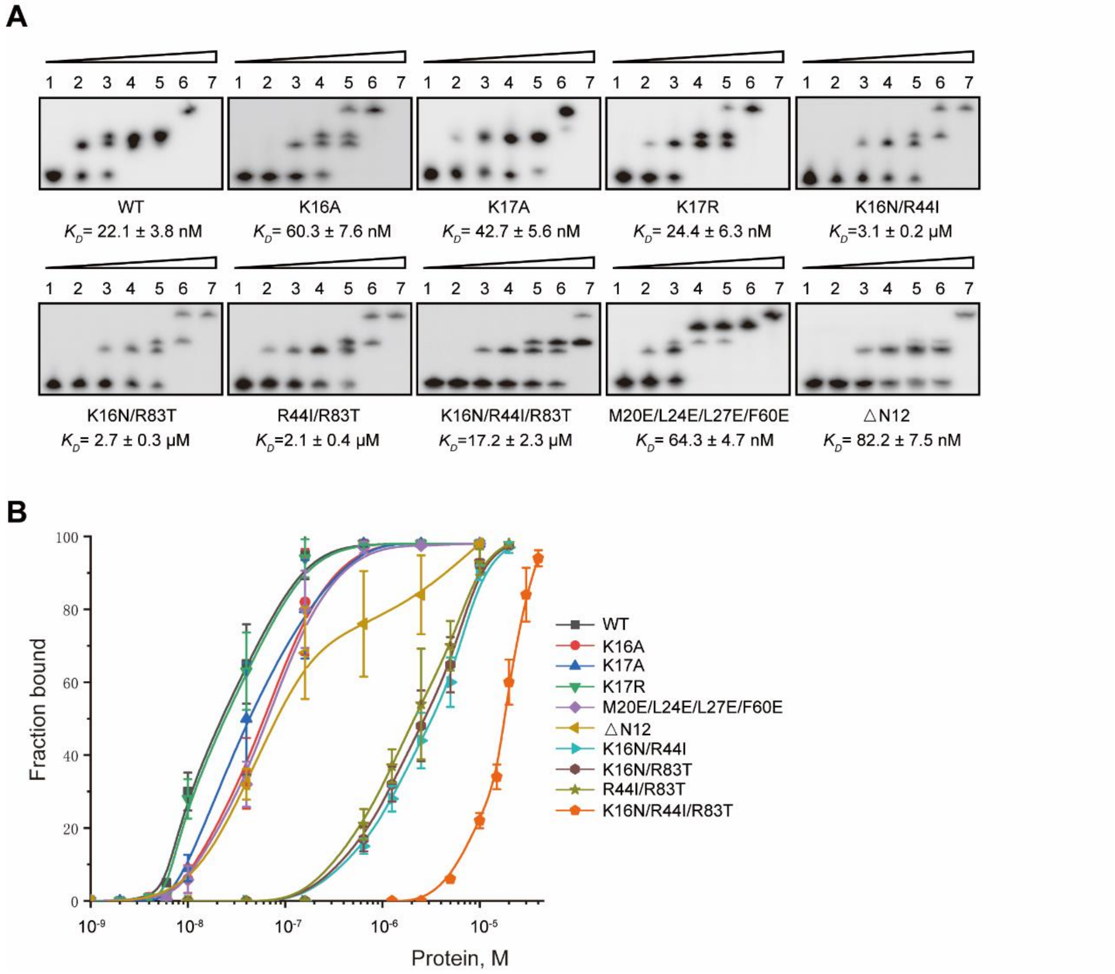

Supplementary Figure S6. RNA binding and oligomerization by wild-type and mutant Sis10b proteins. (A) Analysis of the binding of wild-type and mutant Sis10b to dsRNA by EMSA. Wild-type or mutant Sis10b was incubated with a ^{32}P-labelled 25-bp dsRNA fragment for 30 min at 25°C. Samples were subjected to electrophoresis in a 15% polyacrylamide gel. The gels were exposed to a storage phosphor-imaging screen. For wild-type Sis10b， K16A, K17A, K17R, M20E/L24E/L27E/F60E and △N12, protein concentrations in lanes 1–7 were 0, 0.01, 0.04, 0.16, 0.64, 2.5 and 10 µM, respectively. For K16N/R44I K16N/R83T and R44I/R83T, protein concentrations in lanes 1–7 were 0, 0.64, 1.25, 2.5, 5, 10 and 20 µM, respectively. For K16N/R44I/R83T, protein concentrations in lanes 1–7 were 0, 5, 10, 15, 20, 30 and 40 µM, respectively. Apparent K_D values represent an average of three independent measurements. (B) Quantification of the data in the above EMSA assays. The fraction of the protein-bound RNA was plotted against the concentration of the protein. Apparent K_D was determined by the concentration of the protein at which half of the input RNA was retarded. Each K_D value represents an average of three independent measurements.

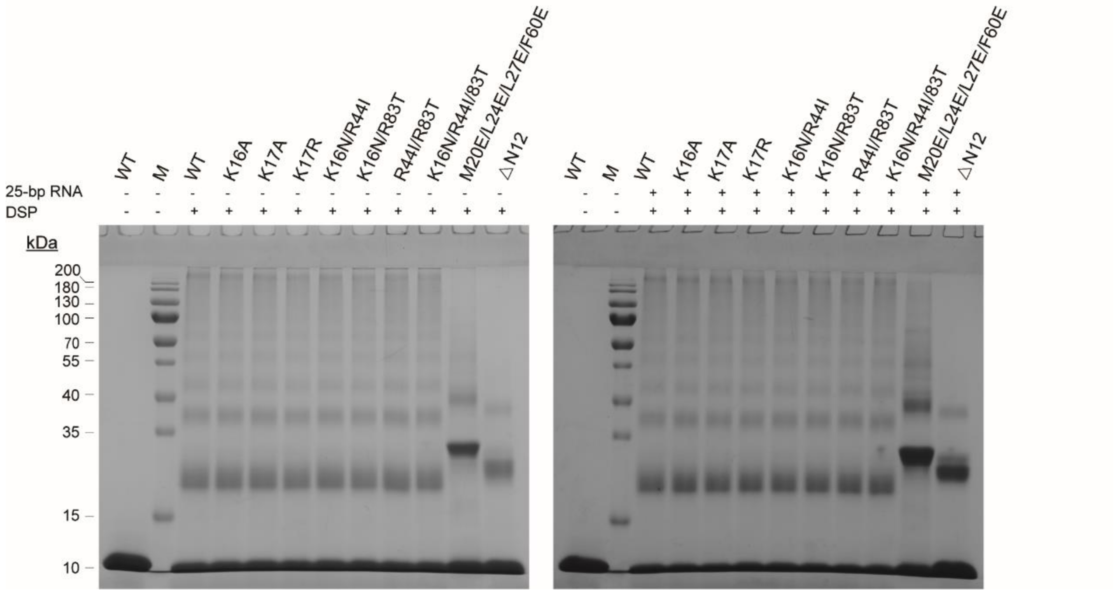

Supplementary Figure S7. Analysis of the ability of wild-type and mutant Sis10b to form oligomers at 25ºC. Wild-type or mutant Sis10b was cross-linked alone (left panel) or in the presence of a 25-bp dsRNA (right panel) with 0.5 mM DSP for 30 min at 25ºC. Samples were analyzed by 15% SDS-PAGE. Molecular mass standards are indicated.

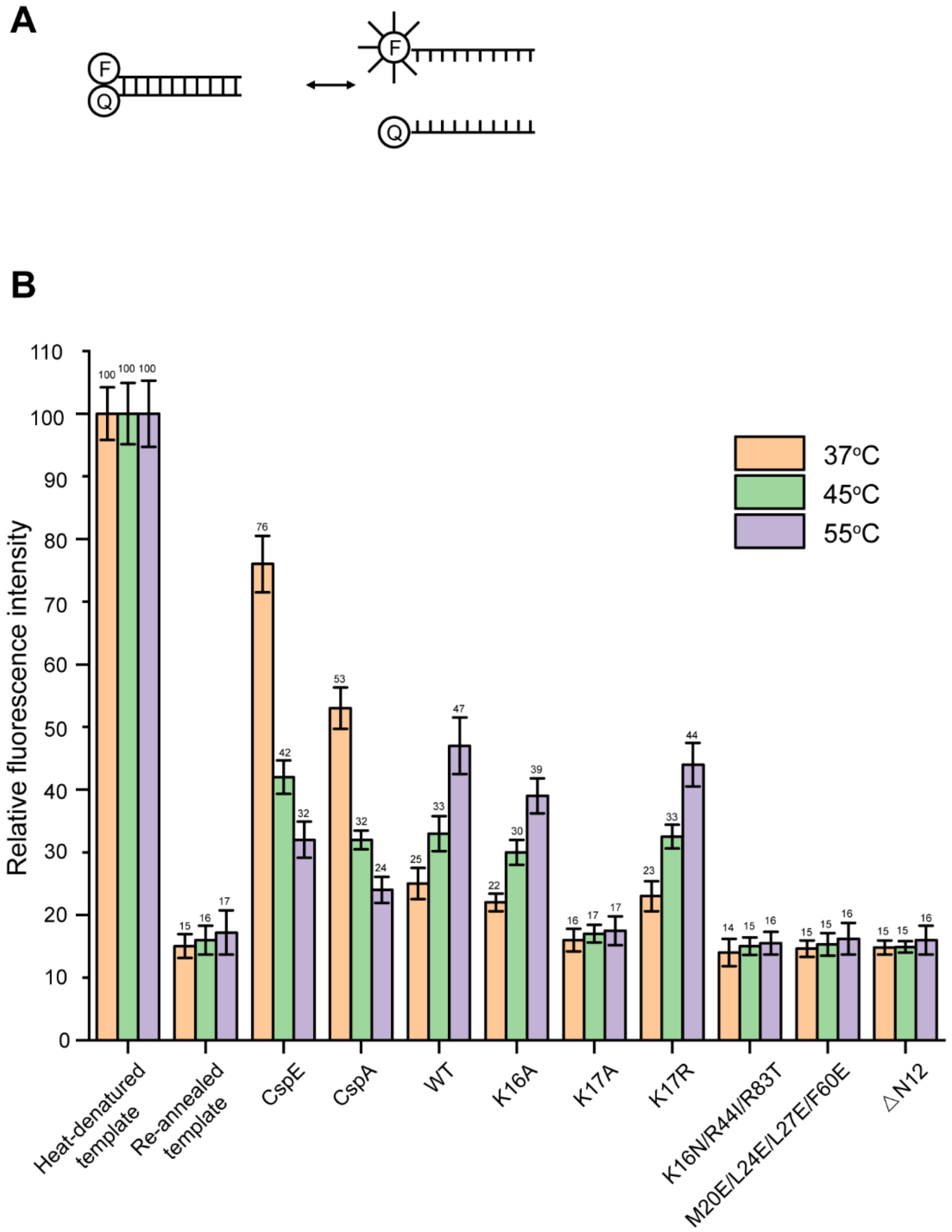

Supplementary Figure S8. Unwinding of blunt-ended duplex RNA by wild-type and mutant Sis10b. (A) A sketch of a FITC-labelled blunt-ended dsRNA template used in unwinding assays. (B) The dsRNA unwinding activities of wild-type and mutant Sis10b. Each protein (20 μM) was mixed with the FITC-labelled template (0.1 μM), and the change in fluorescence was monitored at indicated temperatures. The fluorescence of the heat-denatured FITC-labelled template was defined as 100% and that of the annealed dsRNA template was a control. Each number represents an average of three independent measurements.

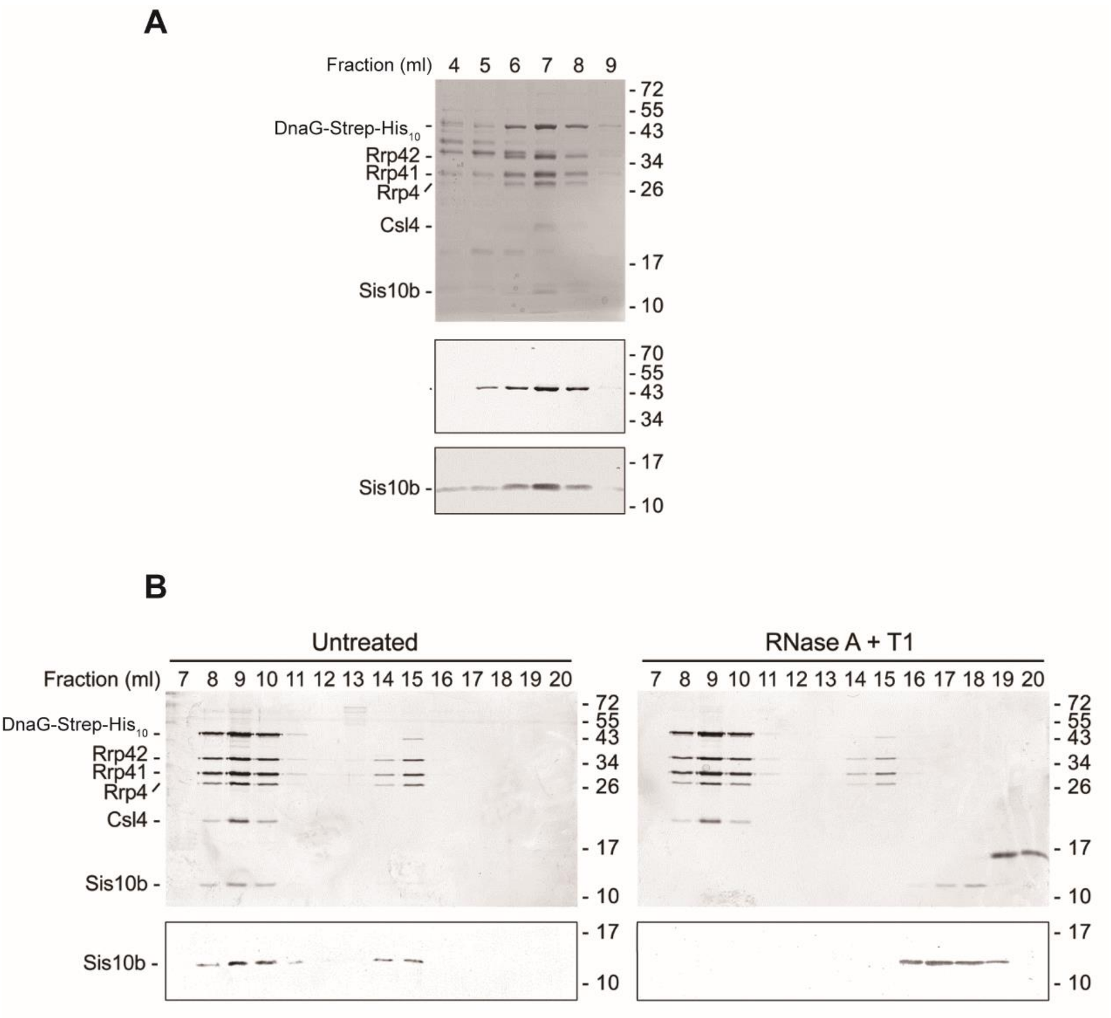

Supplementary Figure S9. Analysis of the association of exosomes and Sis10b. (A) Co-purification of exosome and Sis10b. Cell lysates of *S. islandicus* LH1, a mutant strain that encoding DnaG-Strep-His_{10} hybrid, were subjected to HiTrap chelating column analysis. Aliquots from each fraction were separated by electrophoresis through 15% SDS-PAGE gels and stained with Coomassie brilliant blue or detected by immunoblotting with anti-His or anti-Sis10b antibodies. Upper, SDS-PAGE. Lower, western blot. (B) Detection of the interaction of Sis10b with exosomes. Exosomes containing Sis10b purified under 0.15 M NaCl were untreated or treated with RNase A+T1 and loaded on Superdex 200 column. Fractions were separated by electrophoresis through 15% SDS-PAGE gels and stained with silver (upper) or detected by immunoblotting with anti-Sis10b antibodies (lower).

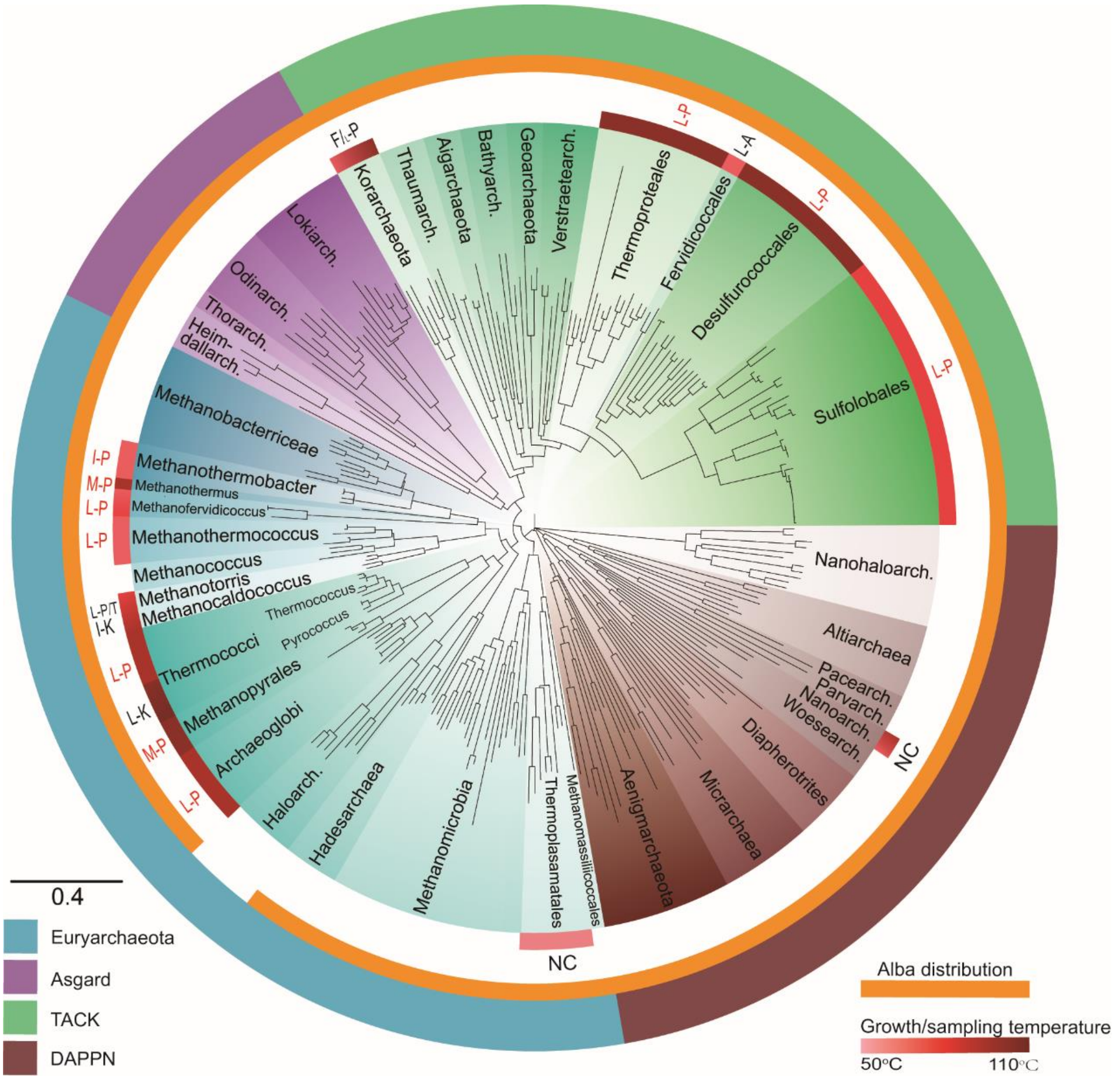

Supplementary Figure S10. A 16S rDNA-based phylogenetic tree showing the distribution of the Sac10b family proteins in Archaea as well as residues corresponding to positions 61 and 62 in Sis10b. The 16S rDNA sequences were retrieved from the NCBI nr database, aligned with MAFFT-LINSi, trimmed with TRIMAL, and analyzed with IQ-TREE. The tree was rooted with the DPANN Archaea. Archaeal superphyla are indicated in the outermost circle. Archaea with an optimal growth or sampling temperature of higher than 50ºC are marked with red arcs (from light to dark red with an increase in temperature). Homologous sequences of the Sac10b protein were identified by PSI-BLAST search and their distribution is indicated in the orange arc. Residues corresponding to positions 61 and 62 in Sis10b were analysed by multi-sequence alignment. Sequences containing a proline residue at position 62 are mainly distributed in thermophilic and hyperthermophilic archaea, and most of these organisms encode at least a Sac10b homolog containing X61-P62 (labelled in red, X stands for L, I or M). Positions equivalent to positions 61 and 62 in Sis10b are not well conserved (NC) in *Thermoplasamatales* and *Nanoarchaeota*. Scale bar indicates the number of substitutions per site.

Supplementary Tables

Supplementary Table S1. Oligonucleotides used in this study[1, 2, 3, 4].

Designation	Sequence (5'–3')
sis10b-NdeI (f)	GGAATTC**CATATG**AGCAGCGGAACCCCAAC
sis10b-XhoI (r)	CCG**CTCGAG**TTACTTTTTCCTTATAGCAATTTCTATTGTTG
sis10b-NotI (r)	ATAAGAAT**GCGGCCGC**TTACTTTTTCCTTATAGCAATTTCTATTGTTG
sis10b-BamHI (r)	CGC**GGATCC**TTACTTTTTCCTTATAGCAATTTCTATTGTTG
K16A (f)	GTAGTCTTAATAGGAGCTAAACCAGTAATGAAC
K16A (r)	GTTCATTACTGGTTTAGCTCCTATTAAGACTAC
K17A (f)	GTCTTAATAGGAAAGGCTCCAGTAATGAACTATG
K17A (r)	CATAGTTCATTACTGGAGCCTTTCCTATTAAGAC
K17R (f)	CATAGTTCATTACTGGTCTCTTTCCTATTAAGAC
K17R (r)	GTCTTAATAGGAAAGAGACCAGTAATGAACTATG
△N12-NdeI (f)	GGAATTC**CATATG**TTAATAGGAAAGAAACCAGTAATGAACTAT
K16N (f)	CTTAATAGGAAATAAACCAGTAATGAACTATGTCTTAGC
K16N (r)	CTGGTTTATTTCCTATTAAGACTACATTACTTGGAGTTG
R44I (f)	CTAGAGGAATAGCTATTAGTAAGGCCGTAGATACTGTG
R44I (r)	CTAATAGCTATTCCTCTAGCTTTGATTACAATTTCGC
R83T (f)	GACGGAACACAATCAAGAGTTTCAACAATAGAAATTG
R83T (r)	CTTGATTGTGTTCCGTCTTGGCTTGTTACTACTTG
Left-arm-SalI (f)	ACGC**GTCGAC**CCCACCACTTCCCAGCTC
Left-arm (r)	GTTAACCGAATAGAGTAAC
Right-arm-NdeI (f)	GGAATTC**CATATG**AGCAGCGGAACCCCAAC
Right-arm-NotI (r)	ATAAGAAT**GCGGCCGC**CTGTCAACGTCTACGTAAGGC
SOE (f)	GTTACTCTATTCGGTTAACATGTTAAACAAGTTAGGTATAC
SOE (r)	GTATACCTAACTTGTTTAACATGTTAACCGAATAGAGTAAC
Flanking primer (f)	GCTAGCTTAGCGTTGAAACCTAAATC
Internal primer (r)	ACCTAACTTGTTTAACATGTTAACCG
dnaG-upstream-SphI (f)	ATAC**GCATGC**AAGGTAATGTAAAGGTG
dnaG-upstream-PstI (r)	ATAA**CTGCAG**TTAGTGGTGGTGATGGTGATGATGGTGGTGATGCTCGAGCTTTTCAAATT<br>GTGGATGACTCCATCCGGAAGAAGAAATAATGTCAGTAAATGTTAATATG
dnaG-downstream-XmaI (f)	TATG**CCCGGG**TTGATGGAGTTATAACTC
dnaG-downstream-EcoRI (f)	ATAT**GAATTC**TTGATATATCATCAGTTTATC
pyrEF-SalI (f)	AGAT**GTCGAC**TACCCCATCAAACTTATGTCTC
pyrEF-BamHI (r)	AGTC**GGATCC**GTACCTACTGGCGTAGTTTAAC
25-bp dsRNA	GGUAAGAGCACCCGACUGCUCUUCC/GGAAGAGCAGUCGGGUGCUCUUACC
Spacer (t)	AAAGAACTTTTTTAGCTTTATATGAATGACTATTTGAGACCAAAT
Spacer (b)	TAGCATTTGGTCTCAAATAGTCATTCATATAAAGCTAAAAAAGTT
$P_{ara\text{-}SD}$ (t)	ATGTTAAACAAGTTAGGTATACTATTTATAAAATAGTTAGGTCATAAAAGTACCCGAG<br>AATGAGGTGAAGCT**CATATG**GAATTCC

$P_{aras\text{-}SD}$ (b)	GGAATTC**CATATG**AGCTTCACCTCATTCTCGGGTACTTTTATGACCTAACTATTTTATA AATAGTATACCTAACTTGTTTAACAT
44-nt ssRNA (t)	UGACUUCCGCGCGUGAGCUCGAUUCCACAUACCCUAAAUCGCUC
44-nt ssRNA (b)	GAGCGAUUUAGGGUAUGUGGAAUCGAGCUCACGCGCGGAAGUCA
MB_{40}-FITC (t)	FITC-AGGGUUCUUUGUGGCGGCGUCAUCUGUGCUUCCCUAUGCA
MB_{40}-BHQ1 (b)	CCGCCGACGACAGUCGCUUGACGCCGCCACAAAGAACCCU-BHQ1
MB_{22}-FITC (t)	FITC-AGGGUUCUUUGUGGCGGCGUCA
MB_{22}-BHQ1 (b)	UGACGCCGCCACAAAGAACCCU-BHQ1
SA (t)	GGACAGUCGAUGCAGGACC
SB (b)	GGUCCUGCAUCGACGAUUU

[1] Forward and reverse primers are marked by f and r, respectively. [2] The restriction sites are in bold face. [3] t, top strand; b, bottom strand. [4] Complementary regions are underlined with dashed lines.

Supplementary Table S2. Percentages of the chloramphenicol-resistant colonies of *E. coli* RL211 strains carrying pINIII, pINIII-*cspA*, pINIII-*cspE* and pINIII-*sis10b*[1].

	pINIII	pINIII-*cspA*	pINIII-*cspE*	pINIII-*sis10b*
37°C	0	35.2 ±6.4	92.2 ±8.1	11.4 ±2.3
42°C	0	44.6 ±7.2	95.0 ±9.0	33.8 ±4.7

[1]*E. coli* RL211 strains carrying vector pINIII, pINIII-*cspA*, pINIII-*cspE* and pINIII-*sis10b* were incubated to an OD_{600} of 0.4 in liquid LB medium containing 100 μg/ml ampicillin and 0.2 mM IPTG. The cultures were diluted and plated onto LB plates containing 100 μg/ml ampicillin and 0.2 mM IPTG in the presence and absence of 30 μg/ml chloramphenicol. The number of colonies on each plate was counted, and the percentage of the chloramphenicol-resistant colonies, calculated from the ratio of the colonies from a culture on a plate with chloramphenicol to those from the same culture on a plate without the antibiotics, was determined. The average of three independent measurements for each strain at an indicated temperature is shown.
